# Nanoparticle‐Mediated TIPE1 mRNA Delivery Enhances Paclitaxel Sensitivity in Triple‐Negative Breast Cancer by Modulating RAB7A Ubiquitination‐Associated Stability and Autophagy

**DOI:** 10.1002/advs.76975

**Published:** 2026-08-06

**Authors:** Wei Hu, Qishuai Chen, Yan Ma, Yang Liu, Xianing Dong, Wenjuan Wei, Jianxin Du, Shusheng Qiu, Maojin Tian, Peiqing Zhao

**Affiliations:** ^1^ Department of Breast Surgery Zibo Central Hospital Affiliated to Binzhou Medical University Zibo China; ^2^ Center of Translational Medicine Zibo Central Hospital Affiliated to Binzhou Medical University Zibo China; ^3^ School of Medical Laboratory Shandong Second Medical University Zibo China; ^4^ Department of Critical Care Medicine Zibo Central Hospital Affiliated to Binzhou Medical University Zibo China; ^5^ Department of Microbiology Laboratory Zibo Municipal Hospital Zibo China

**Keywords:** autophagy, paclitaxel resistance, RAB7A, TNF Alpha‐Induced Protein 8 Like 1, triple‐negative breast cancer

## Abstract

Acquired paclitaxel (PTX) resistance remains a major obstacle in triple‐negative breast cancer (TNBC) treatment. This study investigated TIPE1's role in regulating autophagy and PTX sensitivity and developed ROS‐responsive TIPE1 mRNA‐loaded nanoparticles (TIPE1m NPs) as a therapeutic strategy. PTX‐resistant TNBC cell lines were established, and integrated transcriptomic and proteomic analyses were performed. TIPE1 was downregulated in resistant cells, while higher TNFAIP8L1 expression in public breast cancer cohorts was associated with better survival and improved PTX response. Mechanistically, TIPE1 overexpression was associated with ubiquitination‐related reduction in the stability of the small GTPase RAB7A, leading to impaired autophagic flux, increased ROS accumulation, and enhanced PTX‐induced apoptosis. Conversely, TIPE1 knockdown stabilized RAB7A, enhanced autophagy, and increased PTX tolerance. ROS‐responsive TIPE1m NPs were constructed to restore TIPE1 expression in resistant cells. TIPE1m NPs suppressed autophagy, increased ROS, and enhanced PTX‐induced apoptosis in vitro. In PTX‐resistant xenografts, combined TIPE1m NPs and PTX treatment suppressed tumor growth without obvious systemic toxicity. These findings identify the TIPE1–RAB7A–autophagy axis as a potential therapeutic target and support TIPE1 mRNA delivery as a strategy to overcome PTX resistance in TNBC.

## Graphical abstract

TIPE1m NPs nanoparticles restore TIPE1 expression, promote RAB7A ubiquitination and degradation, suppress autophagic flux, and resensitize paclitaxel‐resistant triple‐negative breast cancer to therapy.

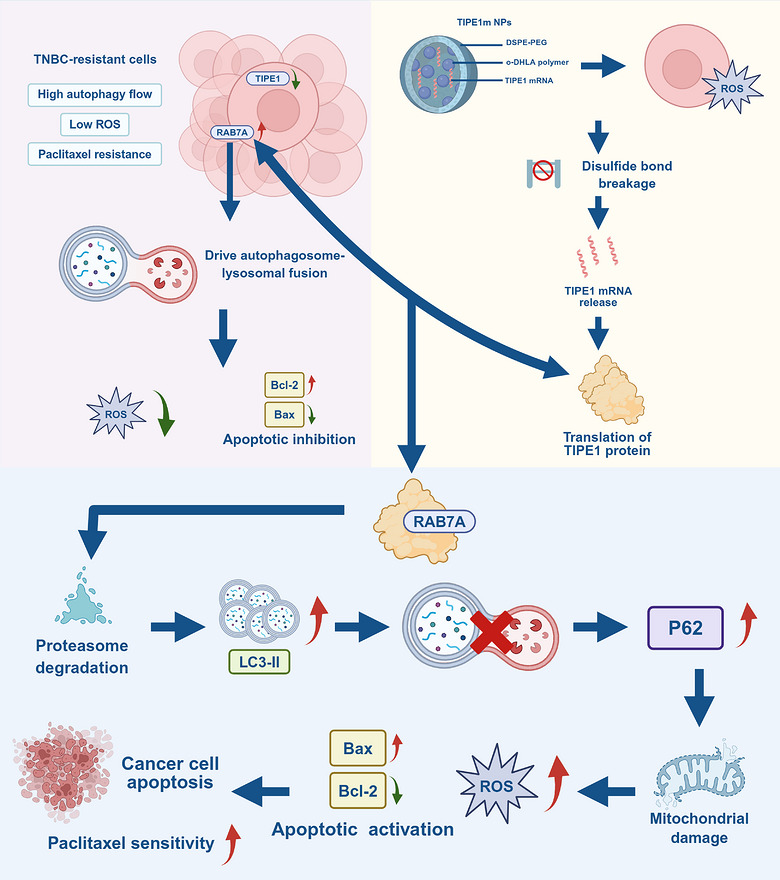

**Abbreviations**
AUC: Area under the Curve
BP: Biological Process
CC: Cellular Component
CCK‐8: Cell Counting Kit‐8
CHX: Cycloheximide
CLSM: Confocal Laser Scanning Microscopy
Co‐IP: Co‐Immunoprecipitation
DHE: Dihydroethidium
DHLA: Dihydrolipoic Acid
DLS: Dynamic Light Scattering
DEGs: Differentially Expressed Genes
DMP: 2,2‐Dimethoxypropane
DMSO: Dimethyl Sulfoxide
EE%: Encapsulation Efficiency
ER: Estrogen Receptor
FBS: Fetal Bovine Serum
FTIR: Fourier‐Transform Infrared Spectroscopy
GO: Gene Ontology
GSEA: Gene Set Enrichment Analysis
H&E: Hematoxylin and Eosin
HER2: Human Epidermal Growth Factor Receptor 2
IHC: Immunohistochemical
LASSO: Least Absolute Shrinkage and Selection Operator
MDC: Monodansylcadaverine
MF: Molecular Function
MMP: Mitochondrial Membrane Potential
NEM: N‐Ethylmaleimide
NMR: Nuclear Magnetic Resonance
o‐DHLA: Oligomeric Dihydrolipoic Acid
oe‐TIPE1: TIPE1 Overexpression
ORF: Open Reading Frame
PI: Propidium Iodide
PR: Progesterone Receptor
PTSA: P‐Toluenesulfonic Acid
PTX: Paclitaxel
RNA‐seq: RNA Sequencing
ROC: Receiver Operating Characteristic
ROS: Reactive Oxygen Species
TEM: Transmission Electron Microscopy
TIPE1: TNF Alpha‐Induced Protein 8‐like 1
TIPE1M NPs: Reactive Oxygen Species‐Responsive TIPE1 mRNA‐Loaded Nanoparticles
TNBC: Triple‐Negative Breast Cancer
WB: Western Blot

## 1 Introduction

Triple‐negative breast cancer (TNBC), defined by the absence of estrogen receptor (ER), progesterone receptor (PR), and human epidermal growth factor receptor 2 (HER2), accounts for approximately 15%–20% of all breast cancer cases [1]. Due to the lack of effective molecularly targeted therapies, chemotherapy remains the main treatment option, with paclitaxel (PTX) widely used as a first‐line regimen [2, 3]. Although many patients initially respond to PTX, acquired resistance frequently develops, leading to reduced therapeutic efficacy and disease progression [4, 5]. Current understanding of the mechanisms underlying PTX resistance remains limited, and effective reversal strategies for clinical application are still lacking [6, 7]. Therefore, identifying the mechanisms driving PTX resistance and developing novel therapeutic targets are critical for improving treatment outcomes in TNBC.

Autophagy is a conserved cellular process that maintains homeostasis by clearing intracellular waste, damaged organelles, and reactive oxygen species (ROS) [8, 9]. In tumor cells, autophagy can alleviate chemotherapy‐induced stress by eliminating drug‐induced ROS, thereby helping cancer cells evade apoptosis and develop drug resistance [10, 11]. Notably, during PTX treatment, autophagy acts as a protective mechanism that enhances ROS clearance and reduces drug cytotoxicity [12, 13]. Conversely, inhibition of autophagic flux, particularly at late stages, promotes ROS accumulation and apoptosis, thereby improving chemosensitivity [14]. Therefore, targeting key regulators of autophagy may represent an effective strategy for overcoming PTX resistance in TNBC.

RAB7A, a small GTPase, is a key mediator of autophagosome–lysosome fusion and is essential for maintaining autophagic flux [15, 16]. Increased RAB7A expression or stability is associated with enhanced autophagy and tumor drug resistance [17]. RAB7A stability can be regulated by ubiquitination, a post‐translational modification controlled by multiple molecular factors [18]. TNF alpha‐induced protein 8‐like 1 (TIPE1), an immune‐ and cell death‐related regulatory protein, is frequently downregulated in various cancers and has been implicated in apoptosis and autophagy regulation [19]. Although TIPE1 may influence cellular responses to chemotherapy, whether it regulates RAB7A ubiquitination and degradation to modulate autophagic flux and drug resistance remains unclear [20]. Clarifying the TIPE1/RAB7A axis may provide a basis for identifying new therapeutic targets.

In this study, we investigated the biological function and molecular mechanism of TIPE1 in PTX‐resistant TNBC, focusing on its involvement in RAB7A ubiquitination‐associated stability regulation, autophagic flux, and ROS accumulation. We further developed ROS‐responsive TIPE1 mRNA‐loaded nanoparticles (TIPE1m NPs) to enhance TIPE1 delivery in resistant tumors with elevated ROS levels. Collectively, this study provides new insights into PTX resistance and supports TIPE1‐based mRNA delivery as a potential therapeutic strategy for overcoming PTX resistance in TNBC.

## 2 Materials and Methods

### 2.1 Cell Culture

MDA‐MB‐231 (HTB‐26) and BT‐549 (HTB‐122) cells were obtained from the American Type Culture Collection (ATCC, USA) and cultured in Dulbecco's modified Eagle's medium (DMEM; 11965‐092, Gibco, USA) supplemented with 10% fetal bovine serum (FBS; 10099‐141, Gibco, USA) and 1% penicillin–streptomycin (15140122, Gibco, USA). Cells were maintained at 37°C in a humidified incubator with 5% CO_2_.

PTX‐resistant MDA‐MB‐231R and BT‐549R cells were established by continuous exposure to gradually increasing concentrations of PTX (T7402, Sigma–Aldrich, USA). The initial PTX concentration was 1 nm and was increased to 10 nm over 6 months until cells stably proliferated in 10 nm PTX [21].

Human embryonic kidney cells (HEK‐293; CL‐0001) were purchased from Wuhan Procell Life Science & Technology Co., Ltd. These cells were cultured in high‐glucose DMEM (11995‐065, Gibco, USA) supplemented with 10% FBS and 1% penicillin‐streptomycin and maintained at 37°C in a 5% CO_2_ atmosphere. HEK‐293T cells were used for lentiviral packaging and plasmid transfection.

### 2.2 Cell Proliferation Assay

Cell viability was determined using a Cell Counting Kit‐8 (CCK‐8; CK04, Dojindo, Japan). Briefly, cells were seeded into 96‐well plates at 5 × 10^3^ cells/well. After 12 h, PTX was prepared as a DMSO stock solution and freshly diluted into culture medium to final concentrations of 1, 2, 5, 10, 20, or 40 nm for 48 h; matched vehicle‐control wells received the same final DMSO exposure as the corresponding PTX concentration series. Cells were then incubated with 10 µL CCK‐8 solution at 37°C for 1 h. Absorbance was measured at 450 nm using a Synergy H1 microplate reader (BioTek, USA), and medium‐only wells were used for background subtraction. All assays were performed with three independent biological replicates and three technical replicates per concentration. IC50 values and 95% confidence intervals were calculated using SPSS 25.0, and all valid biological replicates were included unless model convergence or fit‐quality criteria failed.

### 2.3 Apoptosis Assay

Apoptosis was assessed using an Annexin V‐FITC/propidium iodide (PI) dual‐staining kit (556547, BD Biosciences, USA). Briefly, cells were seeded in six‐well plates and treated with 20 nm PTX for 24 h after attachment. The cells were then collected, washed twice with phosphate‐buffered saline (PBS), and resuspended in Annexin V binding buffer, followed by sequential staining with Annexin V‐FITC and PI. After incubation in the dark for 15 min, samples were analyzed using a BD FACSCanto II flow cytometer (BD Biosciences, USA), and data were quantified with FlowJo software.

### 2.4 RNA Sequencing (RNA‐Seq)

To identify key molecules associated with PTX resistance, transcriptome sequencing was performed on MDA‐MB‐231 (Ctrl, *n* = 3) and MDA‐MB‐231R (Re, *n* = 3) cells. RNA libraries were prepared using the NEBNext Ultra II RNA Library Prep Kit (New England Biolabs, USA), followed by paired‐end 150 bp sequencing on an Illumina NovaSeq 6000 platform (Illumina, USA), generating approximately 50 million reads per sample. Sequencing quality was assessed using FastQC (v0.11.9), and adapters were trimmed using Trimmomatic (v0.39). Reads were aligned to the human reference genome GRCh38 using HISAT2 (v2.2.1), and gene expression was quantified with featureCounts (v2.0.3). Differentially expressed genes (DEGs) were identified using DESeq2 (v1.30.1) with thresholds of false discovery rate (FDR) < 0.05 and |log_2_FoldChange| ≥ 1. Volcano plots were generated in R using ggplot2 (v3.3.5), with log_2_FoldChange on the x‐axis and −log_10_(FDR) on the y‐axis; significantly upregulated and downregulated genes were color‐coded.

### 2.5 Pathway Enrichment and Gene Set Enrichment Analysis (GSEA)

DEGs obtained from DESeq2 were subjected to Kyoto Encyclopedia of Genes and Genomes (KEGG) and Gene Ontology (GO) (Biological Process [BP]/Cellular Component [CC]/Molecular Function [MF]) enrichment analysis using the clusterProfiler package (v3.18), with a significance threshold set at FDR < 0.05. The original gene expression matrix was used for GSEA (v4.1), referencing the MSigDB HALLMARK gene sets. The analysis was performed with 1000 permutations, and significance was defined as |NES| > 1.5 and FDR < 0.25.

### 2.6 Least Absolute Shrinkage and Selection Operator (LASSO) Regression Modeling

LASSO logistic regression was performed in R using the glmnet package (v4.1‐3). The differentially expressed gene expression matrix was used as the input, and binary group labels (0/1) were used as the response variable. Ten‐fold cross‐validation with cv.glmnet was applied to determine the optimal penalty parameter (λ.min), and genes with nonzero coefficients at λ.min were selected as candidate features.

### 2.7 Random Forest Feature Selection

Random forest‐based feature selection was performed in R using the randomForest package (v4.7‐1.1). Candidate genes identified by LASSO regression were used as the input matrix, with group labels as the response variable. The model was built with 500 trees, and mtry was set to the square root of the number of features. Feature importance was evaluated using Mean Decrease Accuracy and Mean Decrease Gini.

### 2.8 Heatmap Visualization

Expression levels (normalized TPM or FPKM) of the LASSO‐selected genes across all samples were visualized using the pheatmap package (v1.0.12). Parameters were set as row_scale = TRUE, with both cluster_rows and cluster_cols enabled.

### 2.9 Receiver Operating Characteristic (ROC) Curve Evaluation

ROC analysis of the candidate gene TNFAIP8L1 was conducted using the pROC package (v1.17.0.1). Gene expression values and group labels were used as inputs. Bootstrapping with 2000 resampling iterations was applied, and the area under the curve (AUC) was calculated to evaluate discriminative performance.

### 2.10 Gene Importance Scatter Plot

A 2D scatter plot was generated in R using the ggplot2 package (v3.3.5), with LASSO regression coefficients and random forest importance scores. The x‐axis represents the Mean Decrease Gini rankings from the random forest model, while the y‐axis corresponds to the absolute values of the LASSO logistic regression coefficients. Key genes located in the upper‐right quadrant were specifically highlighted.

### 2.11 Public Data Acquisition and Expression Analysis

TCGA‐BRCA RNA‐sequencing data were obtained from the UCSC Xena database, including 292 normal breast tissue samples and 1099 breast cancer samples. The expression matrix was downloaded in log_2_(TPM + 1) format. Differential expression of TNFAIP8L1 and RAB7A was analyzed using limma (v3.52.4) in R (v4.2.1), and boxplots were generated with ggplot2 (v3.3.5).

### 2.12 Survival and Treatment Response Analysis

The association between TNFAIP8L1 or RAB7A expression and breast cancer prognosis was assessed using the Kaplan–Meier Plotter database. Patients were stratified into high‐ and low‐expression groups according to the median expression level of each gene. Overall survival (OS) was compared between groups, and hazard ratios (HRs) with Log‐rank *p*‐values were recorded.

The association between gene expression and PTX response was assessed using the ROC Plotter database. Breast cancer patients who received paclitaxel treatment were selected, and gene expression data from responders and non‐responders were extracted. ROC curves were generated, and the area under the curve (AUC) and corresponding *p*‐values were calculated.

### 2.13 Prediction of Ubiquitination Sites and Regulatory Network

RAB7A ubiquitination sites and potential regulatory networks were predicted using public databases. The PhosphoSitePlus database was used to retrieve reported post‐translational modification sites of RAB7A. The UbiBrowser database was used to predict potential E3 ubiquitin ligases and deubiquitinases (DUBs) targeting RAB7A. TNFAIP8L1, RAB7A, and the predicted ubiquitination‐related regulators were then imported into NetworkAnalyst. A protein–protein interaction (PPI) network was constructed using the IntAct Interactome database as the background interaction source, with the degree cutoff set to 5.0 for network filtering and visualization.

### 2.14 TMT‐Based Quantitative Proteomic Analysis

MDA‐MB‐231R negative control overexpression (oe‐NC) and TIPE1 overexpression (oe‐TIPE1) cells were subjected to tandem mass tag (TMT)‐based quantitative proteomic analysis, with three biological replicates per group. Cell pellets were lysed in buffer containing 1% SDS (L3771, Sigma–Aldrich, USA) and protease inhibitor cocktail, and protein concentrations were measured using a BCA protein assay kit (23225, Thermo Fisher Scientific, USA). For each sample, 100 µg protein was digested overnight at 37°C with trypsin at an enzyme‐to‐protein ratio of 1:50. Peptides were labeled using a TMT 6‐plex reagent kit (90061, Thermo Fisher Scientific, USA).

The labeled peptides were separated using a high‐performance liquid chromatography system (Ultimate 3000 RSLCnano, Thermo Fisher Scientific, USA) and analyzed on a Q Exactive HF‐X mass spectrometer (Thermo Fisher Scientific, USA). Raw mass spectrometry files were processed using Proteome Discoverer software (v2.4, Thermo Fisher Scientific, USA) and searched against the UniProt Human database (v2022_01; 20 386 sequences). The FDR was set to 1% for peptide and protein identification. Differentially expressed proteins (DEPs) were defined as those with |log_2_FC| > 0.585 and adjusted *p*‐value < 0.05.

### 2.15 Proteomic Data Analysis

Principal component analysis (PCA) was performed using FactoMineR (v2.4) and factoextra (v1.0.7) in R (v4.2.1) to evaluate the overall distribution of proteomic profiles and sample clustering. KEGG pathway enrichment analysis of DEPs was conducted using clusterProfiler (v4.4.4), with PadjustCutoff set to 0.05. The top 10 enriched pathways were visualized as bubble plots. Quantitative data for Rab family GTPases and autophagy‐related proteins, including ATG5 and BECN1, were visualized and statistically analyzed using ggplot2.

### 2.16 RT‐qPCR

Total RNA was reverse‐transcribed into cDNA using the PrimeScript RT Reagent Kit (RR037A, Takara Bio Inc., Japan). Quantitative PCR was performed using TB Green Premix Ex Taq II (RR820A, Takara Bio Inc., Japan) on an ABI QuantStudio 5 system (Thermo Fisher, USA). Each 20 µL reaction contained 2 µL cDNA, 10 µL TB Green Mix, 0.8 µL of each primer, and ddH_2_O. The cycling conditions were 95°C for 30 s, followed by 40 cycles of 95°C for 5 s and 60°C for 30 s, with melting curve analysis. Relative expression levels of Beclin‐1, ATG5, RAB7A, and TIPE1 were calculated using the ΔΔCt method, with GAPDH as the internal control. Primer sequences are listed in Table S1.

### 2.17 Western Blot (WB)

WB was performed to detect autophagy‐related proteins, TIPE1, and RAB7A. Cells were lysed in RIPA buffer (P0013B, Beyotime, China) supplemented with protease inhibitor cocktail (B15001, Bimake, USA), and protein concentrations were measured using a BCA protein assay kit (23225, Thermo Fisher Scientific, USA). Equal amounts of protein (30 µg/lane) were separated by SDS‐PAGE and transferred onto PVDF membranes (88518, Thermo Fisher Scientific, USA). After blocking with 5% non‐fat milk, membranes were incubated with primary antibodies against LC3B (ab192890, 1:1000, Abcam, UK), Beclin‐1 (ab210498, 1:1000, Abcam, UK), ATG5 (ab108327, 1:1000, Abcam, UK), p‐ULK1 (PA5‐105421, 1:1000, Thermo Fisher Scientific, USA), ULK1 (ULK1‐101AP, 1:1000, Thermo Fisher Scientific, USA), p62 (ab109012, 1:1000, Abcam, UK), TIPE1 (PA5‐103341, 1:1000, Invitrogen, USA), RAB7A (ab137029, 1:1000, Abcam, UK), and GAPDH (ab8245, 1:5000, Abcam, UK). After three TBST washes, membranes were incubated with appropriate HRP‐conjugated secondary antibodies for 1 h at room temperature. Bands were visualized using ECL detection reagent (Bio‐Rad, USA), imaged with a ChemiDoc XRS+ system (Bio‐Rad, USA), and quantified using ImageJ with normalization to GAPDH.

### 2.18 Immunofluorescence Staining

Cells grown on coverslips were fixed with 4% paraformaldehyde (P0099, Beyotime, China) for 15 min, permeabilized with 0.3% Triton X‐100 (T8787, Sigma–Aldrich, USA) for 10 min, and blocked with 1% BSA (A9647, Sigma–Aldrich, USA) for 1 h. Cells were incubated overnight at 4°C with anti‐TIPE1 antibody (PA5‐104229, 1:200, Invitrogen, USA), washed three times with PBS, and then incubated with Alexa Fluor 647‐conjugated goat anti‐rabbit IgG H&L (ab150079, 1:200, Abcam, UK) for 1 h at room temperature in the dark. Nuclei were counterstained with DAPI (ab104139, 1:1000, Abcam, UK), and images were captured using a Zeiss LSM 900 confocal microscope (Zeiss, Germany).

### 2.19 Mutant Construction

The RAB7A‐K191R mutant was generated using the QuickChange XL site‐directed mutagenesis kit (Stratagene, San Diego, CA, USA). The primers used were 5′‐CGAATTTCCTGAACCTATCAGACTGGACAAGAATGACCG‐3′ and 5′‐CGGTCATTCTTGTCCAGTCTGATAGGTTCAGGAAATTCG‐3′. The mutation converted the lysine 191 codon from AAA to AGA, resulting in the K191R substitution. All constructs were verified by sequencing before use in transfection and rescue experiments.

### 2.20 Cell Transfection

TIPE1 knockdown was performed using a short hairpin RNA (shRNA) lentiviral system. Two shRNAs targeting TIPE1 were designed, synthesized, and cloned into the pLKO.1 vector. Recombinant plasmids were co‐transfected into 293T cells with psPAX2 and pMD2.G using Lipofectamine 3000 (L3000008, Invitrogen, USA). Viral supernatants were collected 48 h after transfection and concentrated by ultracentrifugation. MDA‐MB‐231 cells were seeded in six‐well plates and infected with lentivirus in the presence of 8 µg/mL Polybrene (sc‐134220, Santa Cruz Biotechnology, USA). After 24 h, the medium was replaced, and stable cells were selected with 2 µg/mL puromycin (A1113803, Gibco, USA) from 72 h post‐infection. Knockdown efficiency was evaluated by RT‐qPCR and WB, and the most efficient shRNA (sh‐TIPE1) was used for subsequent experiments (Table S2). To assess autophagic flux, stable knockdown cells were treated with 100 nm Bafilomycin A1 (HY‐100558, MedChemExpress, USA) at 37°C for 4 h before further analysis.

For TIPE1 overexpression (oe‐TIPE1), the full‐length TIPE1 cDNA was cloned into the pCDH‐CMV‐MCS‐EF1α‐Puro lentiviral vector. The recombinant plasmid was used to package lentivirus and infect MDA‐MB‐231R cells following the same procedure as the shRNA experiments. Stable overexpression cell lines were selected using puromycin, and TIPE1 overexpression was confirmed by RT‐qPCR and WB.

### 2.21 Colony Formation Assay

Cells were seeded in six‐well plates at 500 cells/well. After 24 h, cells were treated with 20 nm PTX and cultured for 14 days, with medium refreshed every 3 days. Colonies were then washed twice with PBS, fixed with 4% paraformaldehyde for 20 min, and stained with 0.1% crystal violet (C0775, Sigma–Aldrich, USA) for 30 min. Colony images were captured under a microscope, and colony numbers were counted.

### 2.22 Wound Healing Assay

The wound healing assay was used to assess cell migratory ability. Cells were seeded in six‐well plates and grown to approximately 90% confluence. A scratch was made in a straight line using a 200 µL pipette tip. After washing away detached cells with PBS, culture medium containing 2% FBS and 20 nm PTX was added. Cells were incubated for 24 h. Scratch images at 0 and 24 h were captured using an inverted microscope (Olympus, Japan), and wound closure rates were quantified using ImageJ software.

### 2.23 Transwell Invasion Assay

Cell invasion was assessed using Transwell inserts (3422, Corning, USA) coated with Matrigel (356234, Corning, USA) at 37°C for 30 min. Cells (2 × 10^4^) in serum‐free medium were seeded into the upper chamber, and medium containing 10% FBS was added to the lower chamber. After treatment with 20 nm PTX for 24 h, invaded cells were washed three times with PBS, fixed with 4% paraformaldehyde for 20 min, and stained with crystal violet for 30 min. Non‐invading cells were removed with a cotton swab. Five random fields were imaged using an inverted microscope (Olympus, Japan), and invaded cells were counted.

### 2.24 JC‐1 Staining

Cells were seeded in 24‐well plates at 5 × 10^4^ cells/well. After 48 h of PTX treatment, cells were incubated with 2 µm JC‐1 dye (T3168, Thermo Fisher Scientific, USA) at 37°C in the dark for 30 min. Cells were then washed twice with PBS, and red/green fluorescence ratios were observed using a Zeiss LSM 900 confocal microscope (Zeiss, Germany). Changes in mitochondrial membrane potential (MMP) were quantified using ImageJ software.

### 2.25 MitoTracker Red CMXRos Staining

Cells were cultured in glass‐bottom dishes. Following PTX treatment, cells were incubated with 100 nm MitoTracker Red CMXRos (M7512, Thermo Fisher Scientific, USA) at 37°C for 30 min. After three washes with PBS, fluorescence intensity was observed using a Zeiss LSM 900 confocal microscope (Zeiss, Germany) and quantified using ImageJ.

### 2.26 Detection of Intracellular ROS Using DCFH‐DA Probe

Cells were seeded in 24‐well plates at 5 × 10^4^ cells/well. For Mito‐TEMPO treatment, cells were pretreated with 5 µm Mito‐TEMPO (HY‐112879, MedChemExpress, USA) for 1 h before PTX treatment for 48 h. Cells were then incubated with 10 µm DCFH‐DA (D6883, Sigma–Aldrich, USA) at 37°C in the dark for 30 min and washed three times with PBS to remove extracellular probe. Intracellular ROS fluorescence was observed using a fluorescence microscope (Zeiss, Germany), and fluorescence intensity was quantified using ImageJ software.

### 2.27 Monodansylcadaverine (MDC) Staining

Autophagy levels were assessed by MDC staining. Cells were seeded on coverslips in 24‐well plates, allowed to adhere, and incubated in serum‐free medium for the indicated time. Cells were then stained with 50 µm MDC (30432, MERCK, Germany) in 300 µL serum‐free medium at 37°C in the dark for 30 min. After three washes with PBS, cells were fixed with 4% paraformaldehyde (Sigma–Aldrich, USA) for 10 min and washed again with PBS. Nuclei were stained with DAPI (ab104139, 1:1000, Abcam, UK) for 5 min, followed by three PBS washes. Autophagic vacuoles were visualized under DAPI and MDC channels using an inverted fluorescence microscope (Zeiss, Germany), and images were analyzed using Zeiss ZEN software.

### 2.28 TUNEL Staining

Apoptosis in cells and tumor tissues was assessed using TUNEL staining kits (C1086 for cells and C1088 for tissues; Beyotime, China). For cell staining, treated cells were seeded on coverslips, fixed with 4% paraformaldehyde for 20 min, washed with PBS, and permeabilized with 0.1% Triton X‐100 on ice for 5 min. TUNEL staining was then performed according to the manufacturer's instructions, followed by incubation at 37°C in the dark for 1 h. For tissue staining, 4 µm paraffin‐embedded tumor sections from 4% paraformaldehyde‐fixed samples were deparaffinized in xylene and rehydrated through a graded ethanol series. Antigen retrieval was performed using Proteinase K (P1120, Beyotime, China) at 37°C for 15 min, followed by TUNEL staining. For both cell and tissue samples, nuclei were counterstained with DAPI, and TUNEL‐positive cells (green fluorescence) were observed under a fluorescence microscope (Zeiss, Germany). Images were captured using Zeiss Zen software. Five random fields were selected per sample, and the proportion of TUNEL‐positive cells was calculated to quantify apoptotic levels.

### 2.29 LC‐MS/MS Identification of RAB7A and Its Ubiquitination Modifications

Following lysis of HEK‐293 cells, co‐immunoprecipitation (Co‐IP) was performed using an anti‐TIPE1 antibody. The eluted protein complexes were separated by SDS‐PAGE and visualized by silver staining. Specific gel bands were excised, subjected to in‐gel digestion with trypsin (T6567, Sigma–Aldrich, USA), and the resulting peptides were enriched using high‐pH reverse‐phase spin columns. Peptides were separated on a C18 column and analyzed by LC‐MS/MS using an Orbitrap Exploris 480 mass spectrometer (Thermo Fisher, USA). Data were processed with Proteome Discoverer 2.4 software and searched against the UniProt database. Monoisotopic mass errors were calculated, and ubiquitination modification peaks were identified.

### 2.30 Co‐IP Analysis of the Interaction Between TIPE1 and RAB7A

HEK‐293 cells were co‐transfected with HA‐TIPE1 and Flag‐RAB7A plasmids (GeneChem, China). After 48 h, cells were harvested and lysed in NP‐40 buffer (P0013F, Beyotime, China) supplemented with protease inhibitors (B14002, Bimake, USA). For each sample, 1 mg total protein was incubated with HA antibody (3724S, 1:100, Cell Signaling Technology, USA) for 4 h, followed by addition of Protein A/G magnetic beads (88803, Thermo Fisher, USA) and rotation at 4°C for 12 h. The beads were washed three times and boiled in SDS loading buffer. Co‐precipitated proteins were detected by WB using TIPE1 and Flag antibodies (F1804, 1:1000, Sigma–Aldrich, USA).

### 2.31 Cycloheximide (CHX) Chase Assay

Cells were seeded in 6‐well plates and treated with 500 µg/mL CHX (C4859, Sigma‐Aldrich, USA) for 0, 3, 6, or 9 h to inhibit de novo protein synthesis. Total protein was extracted at each time point, and RAB7A degradation was assessed by WB.

### 2.32 Ubiquitination Assay

HEK‐293 cells were co‐transfected with His‐Ub, HA‐TIPE1, and Flag‐RAB7A plasmids (GeneChem, China). After 48 h, cells were treated with 10 µm MG132 (M7449, Sigma–Aldrich, USA) for 6 h to inhibit proteasomal degradation. Cell lysates were supplemented with N‐ethylmaleimide (NEM; E3876, Sigma–Aldrich) to block deubiquitination. Co‐IP was performed using an anti‐RAB7A antibody (ab137029, Abcam), and WB was used to detect ubiquitinated RAB7A (Ub‐RAB7A). MG132 treatment for 6 h further enhanced the observed ubiquitination levels.

### 2.33 mRFP‐GFP‐LC3 Fluorescent Reporter Assay

Cells were transfected with the mRFP‐GFP‐LC3 adenoviral vector (Ad‐mRFP‐GFP‐LC3, Hanbio, China) and incubated for 48 h, followed by treatment with 20 nM PTX for 24 h. Fluorescence signals were observed using a Zeiss LSM 900 confocal microscope (Zeiss, Germany). Red puncta (mRFP^+^/GFP^−^) indicated degraded autolysosomes, while yellow puncta (mRFP^+^/GFP^+^) represented non‐degraded autophagosomes. Fluorescence signals were quantified using ImageJ software.

### 2.34 Synthesis of a ROS‐Responsive Degradable Polymer Based on Oligomeric Dihydrolipoic Acid (o‐DHLA)

A condensation reaction was carried out between dihydrolipoic acid (DHLA; D4586, TCI, China) and 2,2‐dimethoxypropane (DMP; 73137, Sigma–Aldrich, USA). DHLA (206 mg, 1 mmol) and DMP (104 mg, 1 mmol) were dissolved in 20 mL of distilled benzene and transferred to a three‐neck flask equipped with a short‐path distillation head to remove volatile byproducts. The reaction mixture was heated to 95°C under an argon atmosphere. A solution of recrystallized p‐toluenesulfonic acid (PTSA; 0.57 mg, 3 µmol; S580171, Sigma–Aldrich, USA) in distilled ethyl acetate was then added. After 1 h, a solution of DMP in distilled benzene was added dropwise at a rate of 8.67 mg/h. The reaction mixture was stirred overnight, and the final product was obtained by precipitation in cold hexane. The chemical structure of the product was confirmed by nuclear magnetic resonance (NMR; Bruker Avance III, 400 MHz, Germany) and Fourier‐transform infrared spectroscopy (FTIR; Thermo Nicolet iS50, USA).

### 2.35 Preparation of Nanoparticles

TIPE1m NPs were prepared using a self‐assembly method. Briefly, 50 µL of citrate buffer (pH 3.5; P4809, Sigma–Aldrich, USA) was added to 100 µL of G0‐C14 solution (2.5 mg/mL; HY‐152229, MedChemExpress, USA) to facilitate ionization of the cationic lipid. Subsequently, 16 µg of TIPE1 mRNA was added, followed by vortexing for 2 min to form the TIPE1 mRNA/G0‐C14 complex. Then, 50 µL of o‐DHLA (10 mg/mL, dissolved in DMF) was added to the mixture and vortexed for 1 min to generate the lipid‐based nanocomplex. If PEGylation was included in the formulation, DSPE‐PEG was added during the final assembly step to improve colloidal stability and biocompatibility. The TIPE1 mRNA sequence was derived from the predicted transcript of the human TNFAIP8L1 gene (NCBI RefSeq: XM_011527680.3) and contained a full open reading frame (ORF). The mRNA was synthesized by TriLink BioTechnologies (USA) via in vitro transcription. The transcript included a 5′ Cap1 structure and a 3′ poly(A) tail and was unmodified and tag‐free, yielding linear mRNA for subsequent encapsulation into G0‐C14/o‐DHLA‐based nanoparticles.

### 2.36 Characterization of the Physicochemical Properties of Nanoparticles

Particle size and zeta potential were measured by dynamic light scattering (DLS; Zetasizer Ultra, Malvern, UK). Nanoparticle samples were diluted to 1 mg/mL, filtered through a 0.22 µm membrane, and incubated at 37°C before measurement.

For transmission electron microscopy (TEM), 10 µL nanoparticle suspension was dropped onto a carbon‐coated copper grid (Electron Microscopy Sciences, USA). After air‐drying at room temperature, samples were negatively stained and imaged using a Talos F200X transmission electron microscope (Thermo Fisher, USA) to observe morphology.

The encapsulation efficiency (EE%) of TIPE1 mRNA was determined using 5′ Cy5‐labeled TIPE1 mRNA (Cy5‐TIPE1 mRNA; TriLink BioTechnologies, USA). Briefly, 5 µL nanoparticle suspension was mixed with 100 µL dimethyl sulfoxide (DMSO; 94563, Sigma–Aldrich, USA) to dissociate nanoparticles and release encapsulated mRNA. Cy5 fluorescence was measured using a SpectraMax iD5 multimode microplate reader (Molecular Devices, USA) at 650/670 nm excitation/emission wavelengths. Encapsulated mRNA was calculated from a Cy5‐TIPE1 mRNA standard curve, and EE% was calculated as encapsulated mRNA amount/total mRNA amount × 100%.

To assess batch‐to‐batch reproducibility, independently prepared TIPE1m NPs were characterized for particle size, polydispersity index, zeta potential, and encapsulation efficiency, and physicochemical consistency among batches was compared.

### 2.37 ROS‐Triggered Responsiveness of TIPE1m NPs

To evaluate the ROS‐triggered release behavior of TIPE1m NPs, 10 µL of nanoparticle solutions at various concentrations were mixed with 90 µL of PBS (pH 7.4), 1 mm H_2_O_2_, or 10 mm H_2_O_2_ in a 96‐well black plate. After gentle mixing, the plate was placed on a shaker and incubated at room temperature for different time intervals (0, 1, 4, 8, 12, and 24 h). The amount of unbound TIPE1 mRNA in each solution was quantified using the Quant‐iT RiboGreen RNA Assay Kit (R11490, Thermo Fisher Scientific). The release efficiency of TIPE1 mRNA was calculated as the mass ratio of unbound mRNA to the total mRNA content.

In addition, to assess nanoparticle degradation in response to ROS, DLS (Zetasizer Ultra, Malvern, UK) was used to measure particle size after 24‐h incubation with varying concentrations of H_2_O_2_ (0, 1, and 10 mm).

### 2.38 Cellular Uptake and Intracellular Release of Cy5‐Labeled TIPE1M NPs

Cy5‐labeled TIPE1 mRNA (Cy5‐TIPE1 mRNA) was used to prepare Cy5‐TIPE1m NPs for intracellular delivery analysis. Cells were seeded in glass‐bottom dishes at 2 × 10^5^ cells/well and cultured for 24 h, followed by treatment with 100 nm Cy5‐TIPE1m NPs. After incubation, cells were fixed with 4% paraformaldehyde (P0099, Beyotime, China) for 15 min and permeabilized. Lysosomes were stained with LysoTracker Green (C1047S, Beyotime, China) at 37°C for 30 min. Nanoparticle distribution and red/green fluorescence colocalization were observed using a Zeiss LSM 900 confocal microscope (Zeiss, Germany).

### 2.39 In Vivo Pharmacokinetic Analysis

For pharmacokinetic analysis, Cy5‐TIPE1 mRNA‐loaded TIPE1m NPs were administered via tail vein injection at an mRNA‐equivalent dose of 1 mg/kg. Blood samples were collected at 0.5, 1, 2, 4, 8, 12, and 24 h after injection, and plasma was separated by centrifugation at 3000 rpm for 10 min. Plasma Cy5 fluorescence intensity was measured using a fluorescence spectrophotometer (F‐7000, Hitachi, Japan). Fluorescence–time curves were plotted, and the elimination half‐life (t½) was calculated.

### 2.40 Establishment of a Tumor‐Bearing Mouse Model

The antitumor efficacy of TIPE1m NPs combined with PTX was evaluated using an MDA‐MB‐231R xenograft model. Female BALB/c nude mice aged 4–5 weeks were obtained from Beijing Vital River Laboratory Animal Technology Co., Ltd. (strain code: 401, China). Log‐phase MDA‐MB‐231R cells were harvested, resuspended in sterile PBS, mixed with Matrigel at a 1:1 ratio, and subcutaneously injected into the flank of each mouse.

When tumor volume reached approximately 100 mm^3^, mice were randomly assigned to four groups: PBS, PTX, PTX + Blank NPs, and PTX + TIPE1m NPs, with six mice per group. PTX was first dissolved in DMSO and then diluted with sterile PBS immediately before injection, with the final DMSO concentration kept consistent across PTX‐treated groups and the corresponding DMSO‐containing vehicle matched in control injections. Mice in the PTX and combination treatment groups received PTX at 10 mg/kg by intraperitoneal injection twice weekly. Mice in the PTX + Blank NPs and PTX + TIPE1m NPs groups received Blank NPs or TIPE1m NPs, respectively, at an mRNA‐equivalent dose of 400 µg/kg by intravenous injection twice weekly. In the combination treatment groups, PTX was administered intraperitoneally first, whereas Blank NPs or TIPE1m NPs were administered intravenously during the same treatment session after PTX administration. Mice in the PBS group received an equivalent volume of matched vehicle control.

Treatment and monitoring continued for 28 days, during which tumor volume and body weight were recorded regularly. Tumor volume was calculated as V = 0.5 × length × width^2^. At the endpoint, mice were euthanized by intraperitoneal injection of pentobarbital sodium (100 mg/kg), and tumors were excised and weighed. Part of each tumor was fixed in 4% paraformaldehyde for paraffin embedding, while the remaining tissue was stored at −80°C for subsequent protein analysis or histological staining.

Mice were included if tumors were successfully established, tumor volume reached approximately 100 mm^3^, and general health status was acceptable. Before treatment allocation, mice were excluded if tumors failed to form, ulceration or overt infection occurred, body weight decreased by more than 20%, or severe abnormalities unrelated to the intervention were observed. After allocation, all mice meeting the inclusion criteria and completing the procedures were included in the final statistical analysis.

### 2.41 In Vivo Biodistribution Assay

The biodistribution and tumor‐targeting capability of TIPE1m NPs were evaluated in an MDA‐MB‐231R xenograft model. When tumor volume reached 100–200 mm^3^, mice were randomly divided into two groups and intravenously injected with either free Cy5‐labeled TIPE1 mRNA or TIPE1m NPs encapsulating Cy5‐labeled TIPE1 mRNA at an mRNA dose of 400 µg/kg. At 24 h after injection, mice were euthanized, and major organs, including the heart, liver, spleen, lungs, and kidneys, together with tumors, were collected for ex vivo fluorescence imaging using a Syngene PXi imaging system (Synoptics Ltd, UK). Fluorescence intensity was quantified using ImageJ to compare tissue distribution and tumor accumulation.

### 2.42 Dihydroethidium (DHE) Staining

ROS levels in tumor tissues were assessed using DHE (S0063, Beyotime, China). Frozen tumor sections (10 µm) were air‐dried for 10 min, rinsed with PBS, and incubated with 100 µL of 10 µm DHE working solution at 37°C for 30 min in the dark. Sections were then washed three times with PBS, counterstained with DAPI (1:1000), mounted, and observed under a fluorescence microscope (Zeiss, Germany). Red fluorescence indicating ROS‐positive signals was quantified using ImageJ.

### 2.43 Immunohistochemical (IHC) Staining

Mouse tumor tissues were fixed in 4% paraformaldehyde, paraffin‐embedded, and sectioned at 4 µm. After deparaffinization and rehydration, antigen retrieval was performed in 0.01 mol/L sodium citrate buffer (pH 6.0) for 15 min, followed by cooling to room temperature. Endogenous peroxidase activity was blocked with 3% H_2_O_2_ for 10 min, and sections were blocked with 5% bovine serum albumin (BSA) for 30 min. Sections were then incubated overnight at 4°C with anti‐Ki67 (ab15580, 1:200, Abcam, UK) or anti‐TIPE1 (MA5‐27345, 1:100, Invitrogen, USA). The next day, sections were incubated with HRP‐conjugated secondary antibody (PV‐6001, ZSGB‐BIO, China) for 30 min. DAB substrate (34002, Thermo Scientific, USA) was used for chromogenic detection, followed by hematoxylin counterstaining, dehydration, and mounting. Images were captured using a Zeiss microscope, and positive cells were quantified with ImageJ.

### 2.44 Hematoxylin and Eosin (H&E) Staining

After deparaffinization and rehydration, tumor sections were stained with hematoxylin solution (#C0107, Beyotime, China) for 5 min, rinsed with tap water, differentiated in 1% acid alcohol, and blued using bluing solution (#C0109, Beyotime, China). Sections were then stained with eosin Y solution (#C0105, Beyotime, China) for 2 min, dehydrated through graded ethanol solutions (70%, 85%, 95%, and 100%; 1 min each), cleared in xylene for 2 min, and mounted. Tissue morphology was observed and imaged using a Zeiss microscope.

### 2.45 In Vivo Biosafety Evaluation

To evaluate the in vivo biosafety of TIPE1m NPs, body weight, histological examination of major organs, and serum biochemical parameters were assessed at the end of treatment. In addition, a separate 12‐week repeated‐dose study was conducted to evaluate subchronic safety. At the endpoint, major organs and serum samples were collected to assess tissue injury, hepatic and renal dysfunction, and systemic toxicity after prolonged administration. Tissues were then embedded in paraffin, sectioned at 4 µm thickness, and stained with H&E (C0105 and C0107, Beyotime, China). Histological features were examined under a microscope (Carl Zeiss, Germany) to assess the presence of inflammation, necrosis, or other pathological alterations. Blood samples were collected from the retro‐orbital sinus, and serum was separated by centrifugation at 3000 rpm for 10 min. Liver function (ALT, AST) and kidney function (BUN, Cr) markers were analyzed using an automated biochemical analyzer (Hitachi 7600, Hitachi, Japan).

### 2.46 Statistical Analysis

All cell‐based experiments were independently repeated at least three times, and data are presented as mean ± standard deviation (SD). Statistical analyses were performed using GraphPad Prism 9.0 (GraphPad Software, USA). Comparisons between two groups were conducted using unpaired two‐tailed Student's *t*‐tests, while comparisons among multiple groups were analyzed using one‐way analysis of variance (ANOVA) followed by Tukey's post hoc test. Two‐way ANOVA with appropriate multiple‐comparison procedures was used for experiments involving both treatment and time factors. Statistical significance was defined as *p* < 0.05. In animal experiments, mice were randomly assigned to treatment groups after tumor volumes reached the predefined range. Formal randomization was not applied to in vitro experiments. Investigators were not blinded during experimental procedures or outcome assessment. No data point was excluded solely to improve statistical significance; failed nonlinear fits or outliers were handled only according to predefined model‐convergence or quality‐control criteria.

## 3 Results

### 3.1 TIPE1 Expression is Significantly Downregulated in PTX‐Resistant TNBC Cells

**FIGURE 1:**
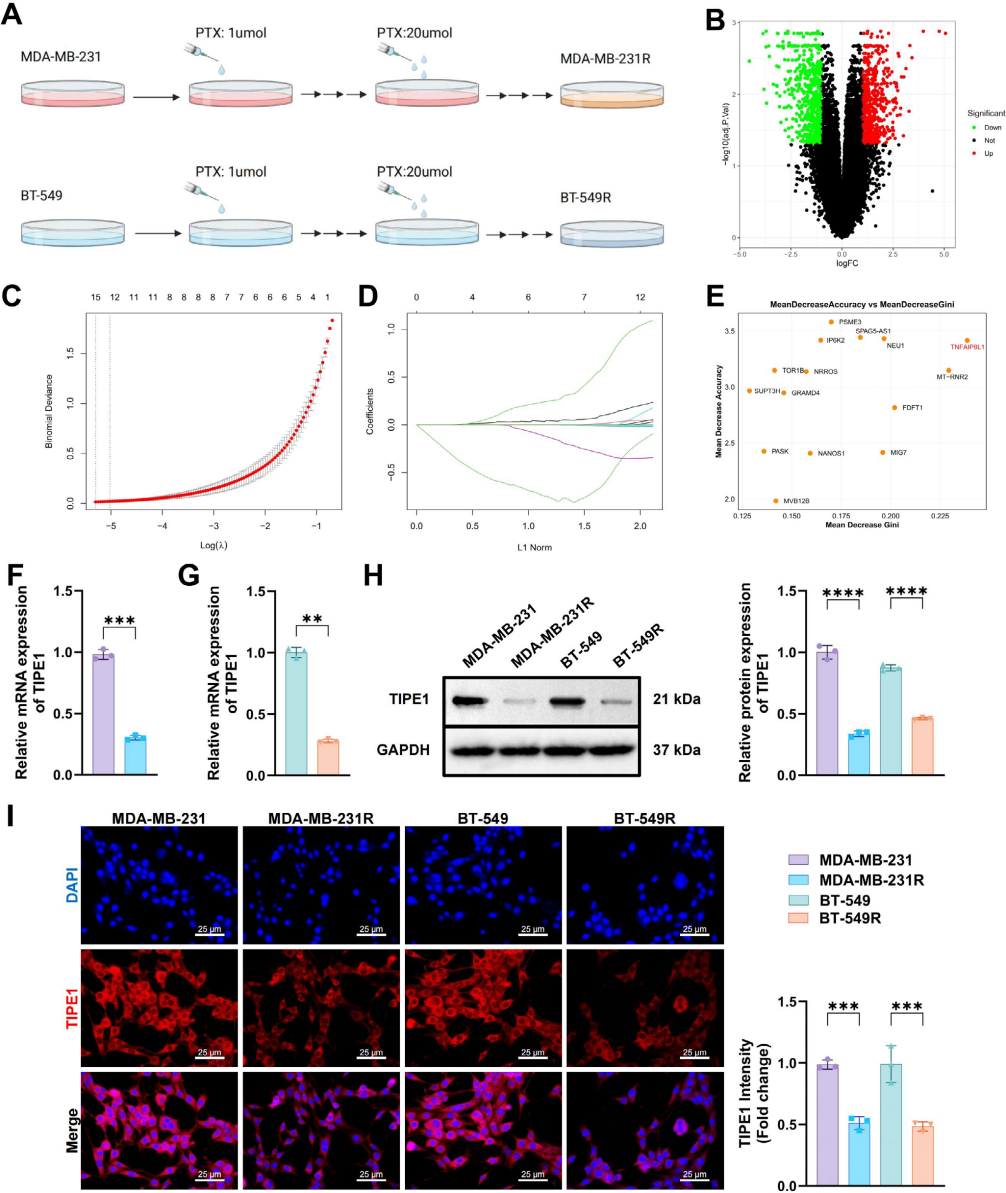
Establishment of PTX‐resistant TNBC cell lines and analysis of TIPE1 expression levels. (A) Schematic illustration of the experimental design showing the cell culture and drug treatment procedures used to generate the MDA‐MB‐231R and BT‐549R resistant cell lines; (B) RNA‐seq analysis identifying DEGs between MDA‐MB‐231 and MDA‐MB‐231R cells; (C) LASSO regression showing the relationship between Log(λ) and mean squared error for resistance‐associated genes; (D) Coefficient trajectories of selected genes in the LASSO model across different λ values; (E) Random forest analysis ranking resistance‐associated genes by importance, based on Mean Decrease Accuracy and Gini index; (F,G) RT‐qPCR analysis of TIPE1 mRNA expression levels in MDA‐MB‐231R and BT‐549R cells; (H) WB analysis of TIPE1 protein levels in MDA‐MB‐231R and BT‐549R cells; (I) Immunofluorescence staining showing the cytoplasmic localization and expression intensity of TIPE1 across TNBC cell groups (Red: TIPE1; Blue: DAPI nuclear stain; scale bar = 25 µm). All data are presented as mean ± SD. Experiments were repeated three times. Statistical analysis was performed using one‐way ANOVA followed by Tukey's multiple comparison test. **p < 0.01, ***p < 0.001, ****p < 0.0001.

To investigate the molecular mechanisms underlying acquired PTX resistance in TNBC cells, we established PTX‐resistant derivatives of MDA‐MB‐231 and BT‐549 cells, designated MDA‐MB‐231R and BT‐549R, respectively. Their resistant phenotypes were then validated by cell viability and apoptosis assays (Figure 1A). CCK‐8 assays showed that, compared with the corresponding parental cells, MDA‐MB‐231R and BT‐549R cells exhibited markedly reduced sensitivity to PTX, as reflected by increased PTX IC50 values (Figure S1A,B). After ten consecutive passages in drug‐free medium, MDA‐MB‐231R and BT‐549R cells still maintained higher PTX IC50 values, suggesting that the resistant phenotype was relatively stable (Figure S1C). Annexin V/PI flow cytometry further showed lower apoptosis rates in resistant cells under the same PTX treatment conditions, indicating enhanced anti‐apoptotic capacity (Figure S1D–F).

To identify resistance‐associated molecules, RNA sequencing was performed on parental MDA‐MB‐231 cells (Ctrl) and MDA‐MB‐231R cells (Re). A total of 1186 DEGs were identified, including 593 upregulated and 593 downregulated genes (Figure 1B). KEGG and GO enrichment analyses showed that these genes were mainly enriched in pathways related to cell cycle, apoptosis, cellular senescence, RNA processing, DNA replication regulation, and transcriptional regulation (Figure S2A–F), suggesting that PTX resistance may involve post‐transcriptional regulation, cell‐cycle remodeling, and altered signal transduction.

GSEA further revealed enrichment of gene sets related to cell‐cycle regulation, DNA damage response, protein homeostasis, and metabolic adaptation in the Re group (Figure S3). HALLMARK_MYC_TARGETS_V1, HALLMARK_G2M_CHECKPOINT, HALLMARK_MITOTIC_SPINDLE, and HALLMARK_E2F_TARGETS were significantly enriched, indicating altered mitosis‐ and proliferation‐related transcriptional programs. Enrichment of HALLMARK_CHOLESTEROL_HOMEOSTASIS and HALLMARK_UNFOLDED_PROTEIN_RESPONSE suggested that lipid metabolism and stress‐adaptive responses may also contribute to PTX resistance.

To screen core resistance‐associated genes, LASSO regression was performed using the DEGs, identifying 15 candidate feature genes at the optimal penalty parameter determined by cross‐validation (Figure 1C,D). Random forest analysis was then used to rank the importance of these 15 genes based on Mean Decrease Accuracy and Mean Decrease Gini (Figure S4A,B). TNFAIP8L1 ranked highly in both metrics, indicating strong discriminatory ability between resistant and non‐resistant cells. The expression heatmap showed that TNFAIP8L1 was markedly downregulated in MDA‐MB‐231R cells (Figure S4C). ROC curve analysis further showed that TNFAIP8L1 had discriminatory performance for the Re group, with an AUC of 0.778 (Figure S4D). In the 2D feature‐importance plot, TNFAIP8L1 was also located in the upper‐right quadrant, further suggesting that it may be an important candidate gene associated with PTX resistance (Figure 1E).

Public database analysis was then used to assess the clinical relevance of TNFAIP8L1. In the TCGA‐BRCA dataset, TNFAIP8L1 expression was significantly higher in breast cancer tissues than in normal breast tissues (Figure S5A). Kaplan–Meier Plotter analysis showed that higher TNFAIP8L1 expression was associated with better OS (Figure S5B). ROC Plotter analysis indicated that TNFAIP8L1 showed modest predictive value for PTX response, with an AUC of 0.609 and a *p*‐value of 4.8 × 10^−2^. Consistently, TNFAIP8L1 expression was higher in responders than in non‐responders (Figure S5C). These results suggest that TNFAIP8L1 is clinically associated with favorable prognosis and improved PTX response.

Finally, we validated the RNA‐seq findings by examining TIPE1, the protein encoded by TNFAIP8L1, in parental and resistant cells. RT‐qPCR showed significantly reduced TIPE1 mRNA levels in MDA‐MB‐231R and BT‐549R cells compared with their parental counterparts (Figure 1F,G). WB further confirmed that TIPE1 protein expression was markedly reduced in both resistant cell lines (Figure 1H). Immunofluorescence staining showed that TIPE1 was mainly localized in the cytoplasm and was visibly weaker in resistant cells (Figure 1I). Collectively, these results demonstrate that TIPE1 is persistently downregulated in PTX‐resistant TNBC cells, suggesting that it may participate in the formation of the PTX‐resistant phenotype.

### 3.2 TIPE1 Enhances PTX Sensitivity in Drug‐Resistant TNBC Cells

**FIGURE 2:**
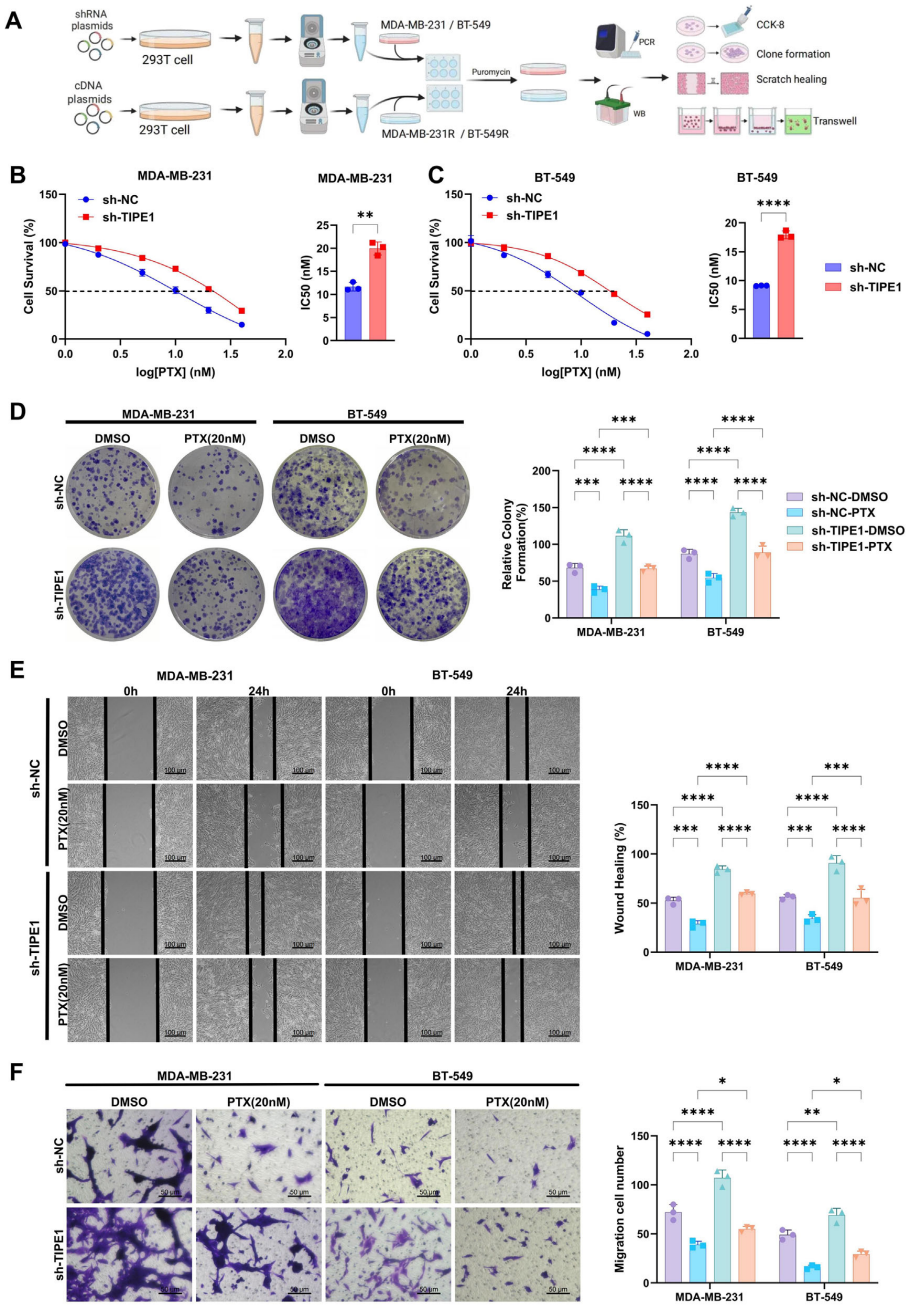
TIPE1 modulates TNBC cell proliferation, migration, and invasion by regulating PTX sensitivity. (A) Schematic of lentiviral infection and antibiotic selection used to establish stable TIPE1 knockdown and overexpression TNBC cell models; (B,C) CCK‐8 assays evaluating changes in PTX sensitivity after TIPE1 knockdown in MDA‐MB‐231 and BT‐549 cells; (D) Colony formation assay assessing proliferative capacity under PTX treatment following TIPE1 knockdown; (E) Wound healing assay analyzing the migratory ability of TIPE1‐deficient TNBC cells under PTX exposure (bar = 100 µm); (F) Transwell invasion assay measuring the invasive capacity of cells after TIPE1 knockdown with PTX treatment (bar = 50 µm). All data are presented as mean ± SD. Experiments were repeated three times. Statistical analysis was performed using ANOVA followed by Tukey's post hoc test. **p < 0.01,***p < 0.001, ****p < 0.0001.

To further investigate the functional role of TIPE1 in PTX resistance in TNBC cells, we silenced TIPE1 (sh‐TIPE1) in MDA‐MB‐231 and BT‐549 cells and oe‐TIPE1 in the drug‐resistant sublines MDA‐MB‐231R and BT‐549R to assess changes in PTX sensitivity, proliferative capacity, and invasiveness (Figure 2A).

We first designed two TIPE1‐targeting shRNA sequences and evaluated knockdown efficiency by RT‐qPCR and WB. The most effective sequence (sh‐TIPE1) was selected for subsequent experiments (Figure S6A,B). CCK‐8 assays showed that TIPE1 knockdown significantly increased the IC_50_ values of MDA‐MB‐231 and BT‐549 cells compared with negative control knockdown (sh‐NC), indicating reduced PTX sensitivity (Figure 2B,C). After 20 nm PTX treatment, sh‐TIPE1 cells also showed increased colony formation relative to sh‐NC cells, suggesting enhanced proliferative capacity (Figure 2D).

Wound healing and Transwell invasion assays further showed that TIPE1 knockdown promoted TNBC cell migration and invasion under PTX treatment. Compared with sh‐NC cells, sh‐TIPE1 cells exhibited a higher wound closure rate and more invading cells across the Matrigel‐coated membrane (Figure 2E,F).

Next, we overexpressed TIPE1 (oe‐TIPE1) in the drug‐resistant MDA‐MB‐231R and BT‐549R cells and evaluated its effects on PTX sensitivity and cellular behavior. CCK‐8 viability assays revealed that compared to the control group (oe‐NC), oe‐TIPE1 significantly reduced PTX tolerance in resistant cells, as evidenced by a marked decrease in IC_50_ values (Figure S7A,B). Moreover, following treatment with 20 nm PTX, the colony formation assay showed a substantial reduction in clonogenic capacity in the oe‐TIPE1 group, indicating that oe‐TIPE1 suppresses the proliferative ability of drug‐resistant TNBC cells (Figure S7C).

We further assessed the effect of oe‐TIPE1 on cell motility and invasion using wound healing and Transwell assays. Following PTX treatment, oe‐TIPE1 cells showed a significantly lower wound closure rate and fewer invading cells than oe‐NC cells, indicating reduced migratory and invasive capacities (Figure S7D,E).

Collectively, these findings demonstrate a positive correlation between TIPE1 expression and PTX sensitivity in TNBC cells. TIPE1 knockdown enhances drug resistance, proliferation, and invasion, whereas oe‐TIPE1 significantly reverses these phenotypes, suggesting a critical role for TIPE1 in mitigating PTX resistance and malignant progression in TNBC.

### 3.3 Knockdown of TIPE1 Promotes Autophagy in TNBC Cells

To investigate the biological characteristics of PTX‐resistant TNBC cells, we first examined their survival strategies. Previous studies have shown that tumor cells can activate autophagy under chemotherapeutic stress to maintain energy homeostasis and mitochondrial integrity, thereby promoting survival [22, 23]. RT‐qPCR and WB showed increased Beclin‐1 and ATG5 expression in MDA‐MB‐231R and BT‐549R cells, accompanied by decreased p62 protein levels, indicating enhanced autophagic flux in resistant cells (Figure S8A–C). JC‐1 and MitoTracker Red CMXRos staining demonstrated that resistant cells exhibited higher MMP and increased mitochondrial content (Figure S8D–F). Additionally, DCFH‐DA staining showed lower intracellular ROS levels in resistant cells compared to their parental counterparts (Figure S8G). Collectively, these results suggest that PTX‐resistant TNBC cells adapt to drug‐induced oxidative stress by enhancing autophagy, maintaining mitochondrial function, and reducing ROS levels (Figure S8H).

**FIGURE 3:**
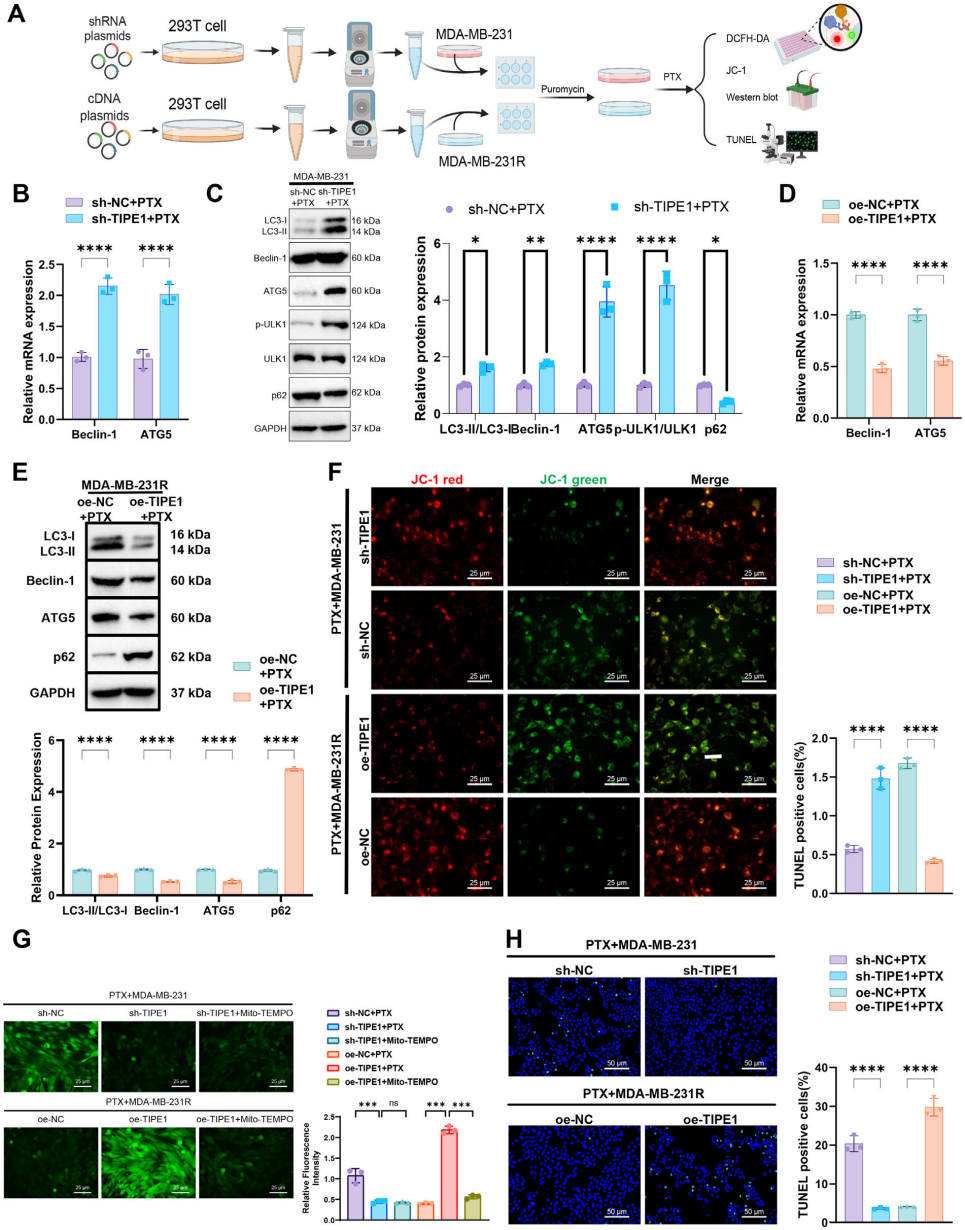
TIPE1 modulates PTX‐induced cytotoxicity by regulating autophagy and mitochondrial function. (A) Experimental workflow illustrating the strategy for assessing changes in autophagy and mitochondrial function following TIPE1 knockdown in MDA‐MB‐231 cells and overexpression in MDA‐MB‐231R cells; (B,C) RT‐qPCR and WB analysis of Beclin‐1 and ATG5 mRNA expression following TIPE1 knockdown in MDA‐MB‐231 cells; (D,E) RT‐qPCR and WB analysis of Beclin‐1, ATG5, and p62 expression after oe‐TIPE1; (F) JC‐1 staining to evaluate changes in MMP following TIPE1 modulation (bar = 25 µm); (G) DCFH‐DA fluorescent probe to detect intracellular ROS levels, including Mito‐TEMPO pretreatment conditions (bar = 25 µm); (H) TUNEL staining to assess apoptosis levels after PTX treatment (bar = 50 µm). All data are presented as mean ± SD. Experiments were performed in triplicate. Statistical significance was determined by ANOVA followed by Tukey's post hoc test. *p < 0.05, **p < 0.01, ***p < 0.001, ****p < 0.0001.

To further define the regulatory role of TIPE1 in this process, TIPE1 was knocked down in parental MDA‐MB‐231 cells and overexpressed in resistant MDA‐MB‐231R cells. Changes in autophagy‐related molecules and mitochondrial function were then evaluated (Figure 3A). TIPE1 knockdown increased Beclin‐1 and ATG5 mRNA levels without significantly affecting ULK1 expression (Figure 3B). Consistently, WB showed that TIPE1 knockdown increased the LC3‐II/LC3‐I ratio and the protein levels of Beclin‐1 and ATG5, while reducing p62 expression; however, it did not further activate ULK1 signaling (Figure 3C). These results suggest that TIPE1 knockdown enhances PTX‐induced autophagy, primarily affecting the downstream autophagic process rather than the upstream initiation stage. In resistant MDA‐MB‐231R cells, TIPE1 overexpression reduced the expression of these autophagy‐related genes (Figure 3D), decreased the LC3‐II/LC3‐I ratio and ATG5 protein level, and increased p62 expression (Figure 3E), indicating that TIPE1 suppresses the autophagic process.

JC‐1 staining revealed that TIPE1 knockdown significantly increased the red/green fluorescence ratio in MDA‐MB‐231 cells, indicating enhanced MMP and improved mitochondrial function. In contrast, oe‐TIPE1 reduced the membrane potential in resistant cells (Figure 3F). DCFH‐DA staining showed that TIPE1 knockdown reduced intracellular ROS levels, whereas TIPE1 overexpression increased ROS accumulation. To further assess whether mitochondrial ROS contributed to the ROS changes induced by TIPE1 overexpression, cells were treated with the mitochondria‐targeted antioxidant Mito‐TEMPO. Pretreatment with 5 µm Mito‐TEMPO for 1 h markedly attenuated the increase in ROS levels induced by TIPE1 overexpression (Figure 3G). These results suggest that TIPE1 may affect intracellular ROS homeostasis and that mitochondrial ROS‐associated changes are involved in TIPE1‐mediated regulation of the PTX response. Furthermore, TUNEL staining demonstrated that TIPE1 knockdown significantly reduced PTX‐induced apoptosis in MDA‐MB‐231 cells, whereas its overexpression increased the number of TUNEL‐positive cells in MDA‐MB‐231R cells (Figure 3H). These findings indicate that TIPE1 enhances PTX cytotoxicity by suppressing autophagy and mitochondrial function, thereby promoting ROS accumulation.

We further examined whether autophagy contributes to the effects of TIPE1 knockdown. Compared with sh‐NC cells, sh‐TIPE1 cells showed enhanced autophagy‐related changes (Figure S9A–C). After Bafilomycin A1 treatment, the LC3‐II/LC3‐I ratio further increased with p62 accumulation, suggesting inhibition of late‐stage autophagic degradation (Figure S9A). MDC staining showed altered autophagy‐associated fluorescence signals after Bafilomycin A1 treatment (Figure S9B), and JC‐1 staining indicated that Bafilomycin A1 partially affected the MMP changes induced by TIPE1 knockdown (Figure S9C). In CCK‐8 assays, TIPE1 knockdown increased the PTX IC_50_ value in MDA‐MB‐231 cells, whereas Bafilomycin A1 abolished the difference between sh‐TIPE1 and sh‐NC cells under PTX treatment (Figure S9D). These results support the involvement of autophagy in TIPE1 knockdown‐induced PTX tolerance.

Together, these findings suggest that TIPE1 may influence PTX sensitivity in TNBC cells by modulating autophagy and mitochondrial status, supporting the potential value of restoring TIPE1 expression as a strategy to overcome PTX resistance.

### 3.4 TIPE1 Promotes the Ubiquitin‐Mediated Degradation of RAB7A

We hypothesized that TIPE1 may participate in the regulation of PTX response in TNBC cells by affecting RAB7A stability and the autophagic process. Public database and bioinformatic analyses were therefore performed to evaluate the clinical relevance of RAB7A and its potential ubiquitination‐related regulatory features. Analysis of the TCGA‐BRCA dataset showed that RAB7A expression was higher in breast cancer tissues than in normal breast tissues (Figure S10A). Kaplan–Meier Plotter analysis indicated that high RAB7A expression was associated with poorer survival outcomes (Figure S10B). ROC Plotter analysis showed a modest association between RAB7A expression and PTX response, with an AUC of 0.561 and a *p*‐value of 2.4 × 10^−^^2^ (Figure S10C).

Further analysis indicated that RAB7A contains several potential ubiquitination sites, including sites near K191/K194 (Figure S10D). UbiBrowser predicted several ubiquitination‐related enzymes that may regulate RAB7A, including SMURF1, SMURF2, NEDD4, HERC3, UBE3C, and STUB1 (Figure S10E). Protein–protein interaction network analysis using NetworkAnalyst showed a connection between TNFAIP8L1 (TIPE1) and the RAB7A‐related regulatory network (Figure S10F). These findings suggest that RAB7A has clinical relevance in breast cancer and that its protein stability may be influenced by ubiquitination‐related regulatory networks, providing a basis for further investigation of the TIPE1–RAB7A regulatory relationship.

**FIGURE 4:**
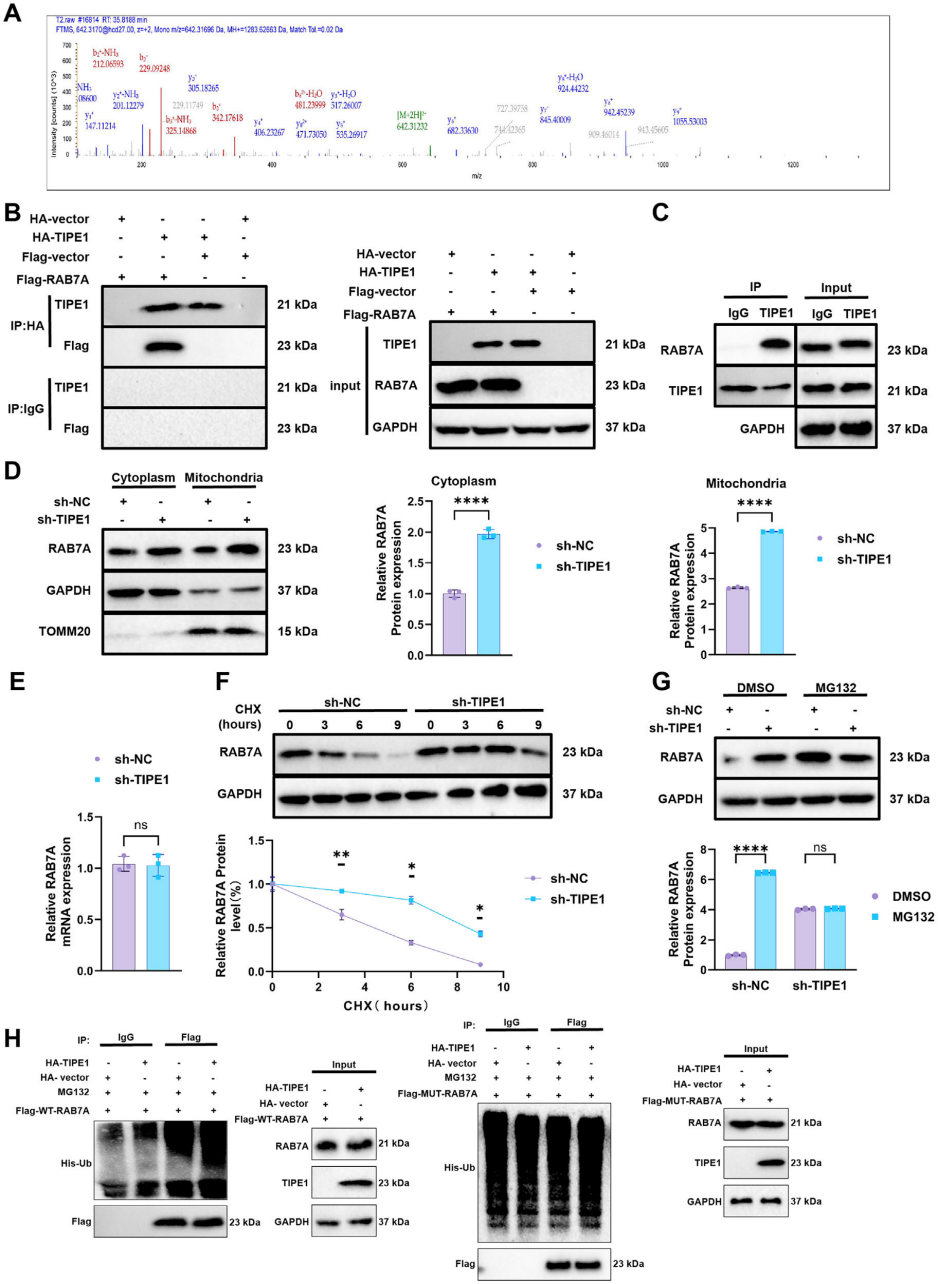
TIPE1 is associated with RAB7A ubiquitination‐related stability regulation and autophagic flux inhibition. (A) LC‐MS/MS analysis showing MS/MS fragmentation spectra of representative RAB7A peptides identified in the TIPE1 immunocomplex; (B,C) Co‐IP assays demonstrating the interaction between TIPE1 and RAB7A in HEK‐293 and MDA‐MB‐231 cells; (D) Subcellular fractionation followed by WB analysis assessing RAB7A distribution in cytosolic and mitochondrial compartments upon TIPE1 modulation; (E) RT‐qPCR analysis of RAB7A mRNA levels following TIPE1 knockdown; (F) CHX chase assay evaluating the stability of RAB7A protein after TIPE1 interference; (G) WB analysis of RAB7A protein levels after proteasome inhibitor MG132 treatment; (H) Co‐IP analysis of RAB7A ubiquitination levels upon HA‐TIPE1 overexpression. All data are presented as mean ± SD. Experiments were repeated in triplicate. Statistical significance was determined by ANOVA followed by Tukey's post hoc test. *p < 0.05, **p < 0.01, ***p < 0.001, ****p < 0.0001; ns: not significant.

Using LC‐MS/MS, we identified the NNIPYFETSAK peptide segment of RAB7A with a monoisotopic mass of 1283.6266 Da, which closely matched the theoretical value (mass error < 0.02 Da). The MS/MS fragmentation spectrum revealed a complete series of b/y ions (e.g., b2^+^ = 229.09, y6^+^ = 942.45) (Figure 4A), confirming the presence of RAB7A in the TIPE1 immunocomplex. Additionally, a partially modified peak (b5‐NH3^+^ = 325.15) suggested that RAB7A may undergo ubiquitination.

To verify whether TIPE1 interacts with RAB7A, Co‐IP assays were performed in HEK‐293 and MDA‐MB‐231 cells. In HEK‐293 cells, RAB7A was markedly enriched in the HA‐TIPE1 immunoprecipitate (IP: HA), whereas no enrichment was observed in the control group (Figure 4B), indicating a potential direct interaction between TIPE1 and RAB7A. Similarly, in MDA‐MB‐231 cells, RAB7A was detected in the endogenous TIPE1 immunocomplex (IP: TIPE1) (Figure 4C), further supporting their interaction.

To investigate whether TIPE1 regulates RAB7A via protein degradation pathways, we examined RAB7A degradation through both proteasomal and autophagic mechanisms. WB showed that RAB7A was markedly increased in both mitochondrial and cytoplasmic fractions of TIPE1‐knockdown cells (Figure 4D), whereas RAB7A mRNA levels were unchanged (Figure 4E), indicating that RAB7A is primarily regulated post‐translationally through protein degradation rather than at the transcriptional level.

A CHX chase assay was conducted to assess RAB7A protein stability further. Following CHX treatment (500 µg/mL), the half‐life of RAB7A in the sh‐NC group was approximately 3 h, whereas it was markedly extended to over 6 h in the sh‐TIPE1 group (Figure 4F), supporting that TIPE1 is associated with reduced RAB7A protein stability. Upon treatment with the proteasome inhibitor MG132, RAB7A accumulated significantly in the sh‐NC group but remained essentially unchanged in the sh‐TIPE1 group (Figure 4G), suggesting that RAB7A may be regulated through a proteasome‐related pathway.

To further determine whether TIPE1 regulates RAB7A stability through ubiquitination, HEK‐293 cells were co‐transfected with HA‐TIPE1, His‐Ub, and Flag‐tagged wild‐type RAB7A (Flag‐WT‐RAB7A) or the RAB7A‐K191R mutant (Flag‐MUT‐RAB7A). RAB7A ubiquitination was then examined by Co‐IP. After MG132 treatment, ubiquitinated Flag‐WT‐RAB7A was markedly increased in cells co‐transfected with HA‐TIPE1. In contrast, a comparable increase in ubiquitinated RAB7A was not observed in cells expressing Flag‐MUT‐RAB7A (Figure 4H). These results suggest that TIPE1 is associated with increased RAB7A ubiquitination and that the K191 site may contribute to this process. The accumulation of Ub‐RAB7A after MG132 treatment further supports the involvement of a proteasome‐related pathway in RAB7A degradation. Together, these findings suggest that TIPE1 may reduce RAB7A protein stability through ubiquitination‐associated regulation, thereby contributing to the regulation of autophagy and PTX response in TNBC cells.

### 3.5 TIPE1 is Associated With RAB7A Ubiquitination‐Related Stability Regulation, Autophagic Flux Inhibition, and Enhanced PTX‐Induced Apoptosis

**FIGURE 5:**
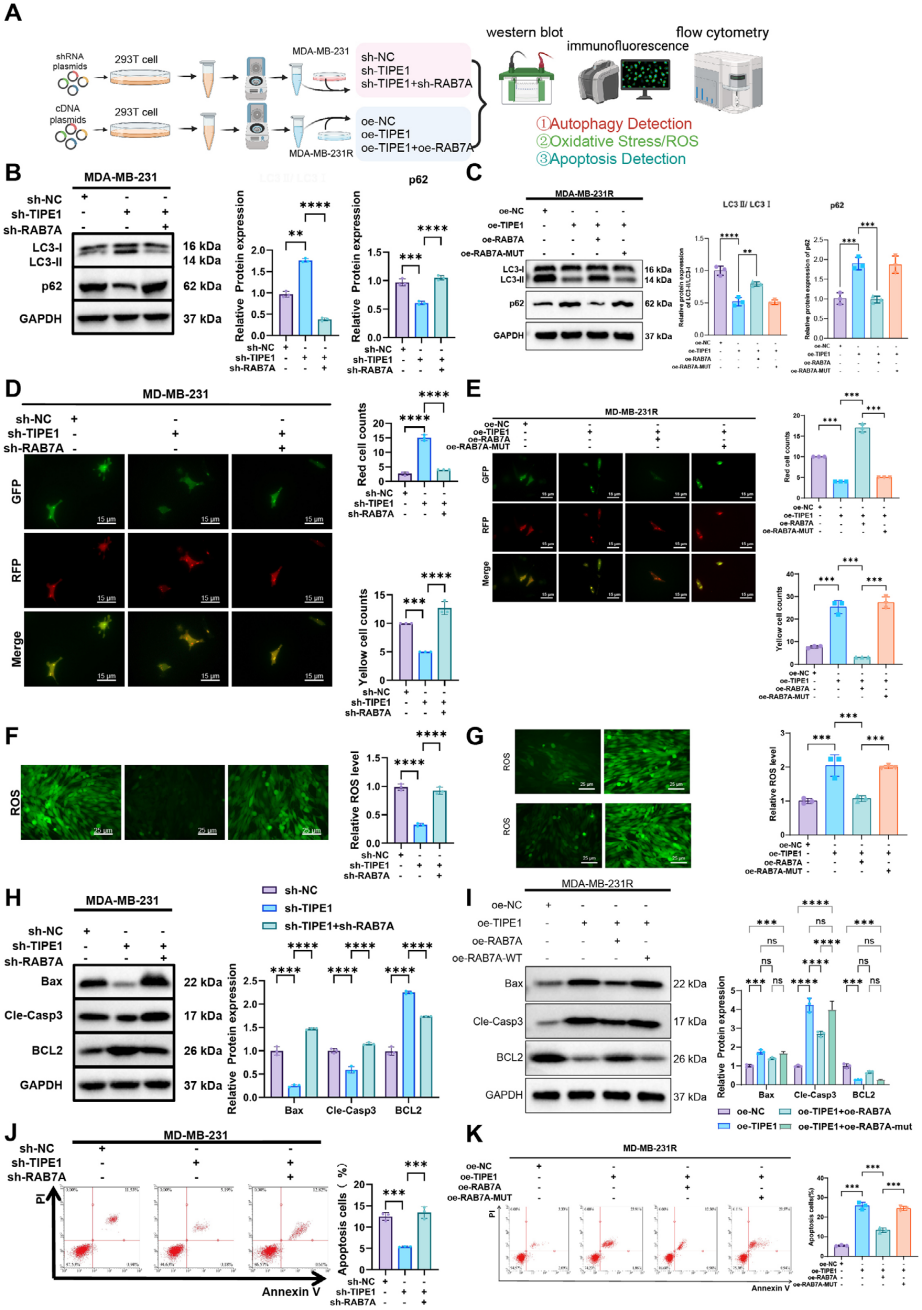
TIPE1 enhances PTX‐induced apoptosis by modulating RAB7A‐mediated autophagic flux blockade and ROS accumulation. (A) Schematic workflow illustrating the experimental strategy for evaluating autophagy, ROS, and apoptosis following manipulation of TIPE1 and RAB7A expression in MDA‐MB‐231 and MDA‐MB‐231R cells; (B,C) WB analysis of LC3‐II/LC3‐I ratio and p62 protein levels after TIPE1 and RAB7A modulation; (D,E) Fluorescence microscopy of mRFP‐GFP‐LC3 dual‐reporter system to assess autophagosome‐lysosome fusion (scale bar = 15 µm); (F,G) DCFH‐DA staining to evaluate intracellular ROS levels following TIPE1 and RAB7A intervention (scale bar = 25 µm); (H,I) WB analysis of cleaved caspase‐3, Bax, and Bcl‐2 to assess apoptosis; (J,K) Annexin V/PI flow cytometry analysis of apoptotic cell populations. All data are presented as mean ± SD. Experiments were independently repeated three times. Statistical significance was determined using ANOVA followed by Tukey's post hoc test. *p < 0.05, **p < 0.01, ***p < 0.001, ****p < 0.0001.

To determine whether TIPE1 affects autophagy through RAB7A, TIPE1 and RAB7A expression were manipulated in MDA‐MB‐231 and MDA‐MB‐231R cells. A RAB7A‐K191R mutant was also included to evaluate the contribution of this site to RAB7A‐mediated regulation (Figure 5A). In MDA‐MB‐231 cells, TIPE1 knockdown increased the LC3‐II/LC3‐I ratio and decreased p62 expression, indicating enhanced autophagy‐related activity. Concurrent RAB7A knockdown reduced the LC3‐II/LC3‐I ratio and partially restored p62 expression, suggesting that RAB7A contributes to TIPE1 knockdown‐induced autophagy changes (Figure 5B). In MDA‐MB‐231R cells, oe‐TIPE1 decreased the LC3‐II/LC3‐I ratio and increased p62 expression, indicating suppression of autophagic flux. Co‐expression of RAB7A partially reversed these effects, whereas the RAB7A‐K191R mutant failed to produce a comparable rescue effect, suggesting that K191 is involved in RAB7A‐mediated autophagy regulation (Figure 5C).

Autophagosome‐lysosome fusion was further evaluated using the mRFP‐GFP‐LC3 reporter. In MDA‐MB‐231 cells, TIPE1 knockdown increased red puncta, whereas combined RAB7A knockdown increased yellow puncta, indicating that RAB7A depletion attenuated TIPE1 knockdown‐induced autophagic flux activation (Figure 5D). In MDA‐MB‐231R cells, oe‐TIPE1 increased yellow puncta, suggesting impaired late‐stage autophagic degradation. RAB7A co‐expression increased red puncta and reduced yellow puncta, whereas the RAB7A‐K191R mutant did not induce similar changes (Figure 5E). These findings further support the involvement of RAB7A in TIPE1‐mediated autophagy regulation.

Because autophagy is closely linked to mitochondrial homeostasis and ROS clearance, intracellular ROS levels were assessed by DCFH‐DA staining. TIPE1 knockdown reduced ROS levels in MDA‐MB‐231 cells, whereas concurrent RAB7A knockdown partially restored ROS accumulation (Figure 5F). Conversely, TIPE1 overexpression increased ROS levels in MDA‐MB‐231R cells. Co‐expression of RAB7A reduced ROS levels, whereas the RAB7A‐K191R mutant did not show a similar effect (Figure 5G). These results suggest that TIPE1 may influence intracellular ROS homeostasis through RAB7A‐associated autophagy regulation.

Apoptosis‐related proteins were then examined after PTX treatment. In MDA‐MB‐231 cells, the sh‐TIPE1 group showed decreased cleaved caspase‐3 and Bax expression and increased Bcl‐2 expression, suggesting reduced apoptosis. Concurrent RAB7A knockdown partially reversed these changes (Figure 5H). In MDA‐MB‐231R cells, oe‐TIPE1 increased cleaved caspase‐3 and Bax expression and decreased Bcl‐2 expression, whereas RAB7A co‐expression partially attenuated these effects. The RAB7A‐K191R mutant did not show a comparable rescue effect (Figure 5I).

Annexin V/PI flow cytometry further supported these findings. In MDA‐MB‐231 cells, TIPE1 knockdown reduced the apoptosis rate, whereas concurrent RAB7A knockdown increased apoptosis. In MDA‐MB‐231R cells, TIPE1 overexpression increased apoptosis, while RAB7A co‐expression reduced the apoptosis rate. In contrast, the RAB7A‐K191R mutant did not lead to a comparable reduction in apoptosis (Figure 5J,K).

Together, these results suggest that TIPE1 may regulate autophagy by affecting RAB7A ubiquitination‐related stability and may thereby contribute to the modulation of ROS homeostasis and PTX‐induced apoptosis. Restoration of RAB7A partially alleviated TIPE1 overexpression‐induced autophagy suppression and apoptosis enhancement, whereas the K191 site may be important for RAB7A‐mediated regulation in this context.

### 3.6 TIPE1 Overexpression Reshapes the Proteomic Profile of Resistant TNBC Cells and Affects RAB7A‐Associated Autophagy‐Related Pathways

To evaluate the global proteomic effects of TIPE1 overexpression in resistant TNBC cells, TMT‐based quantitative proteomic analysis was performed in MDA‐MB‐231R oe‐NC and oe‐TIPE1 cells. A total of 6842 proteins were identified, among which 6317 proteins were reliably quantified. PCA showed clear separation between the oe‐TIPE1 and oe‐NC groups, suggesting that TIPE1 overexpression altered the proteomic profile of resistant TNBC cells (Figure S11A). Differential expression analysis identified 105 DEPs, including 51 upregulated and 54 downregulated proteins (Figure S11B).

Among these DEPs, RAB7A was significantly decreased in the oe‐TIPE1 group, consistent with the CHX chase and ubiquitination assays indicating reduced RAB7A protein stability after TIPE1 overexpression (Figure S11C). Autophagy‐ and endolysosomal trafficking‐related proteins were also altered, with reduced expression of ATG5 and BECN1, suggesting that TIPE1 overexpression may affect protein networks involved in autophagy and vesicular transport (Figure S11D). KEGG enrichment analysis showed that downregulated proteins were mainly enriched in pathways related to Phagosome, SNARE interactions in vesicular transport, Autophagy, Endocytosis, and Lysosome biogenesis, whereas upregulated proteins were enriched in Apoptosis and p53 signaling pathways (Figure S11E).

Further analysis of quantified Rab family small GTPases showed that RAB7A was markedly decreased in the oe‐TIPE1 group with statistical significance (*p* < 0.001). Although several Rab members, such as RAB35, RAB5B, and RAB5A, showed modest changes, most Rab proteins, including RAB1B, RAB10, and RAB14, did not exhibit significant differences (Figure S11F). These findings suggest that TIPE1 overexpression has a prominent effect on RAB7A and autophagy/endolysosomal‐related pathways, without inducing broad coordinated alterations across the Rab family.

Together, the TMT‐based proteomic results provide global protein‐level support that TIPE1 overexpression is associated with RAB7A reduction, remodeling of autophagy‐related protein networks, and activation of apoptosis‐related pathways, further supporting the involvement of the TIPE1–RAB7A axis in PTX resistance regulation.

### 3.7 Successful Construction of TIPE1M NPs

**FIGURE 6:**
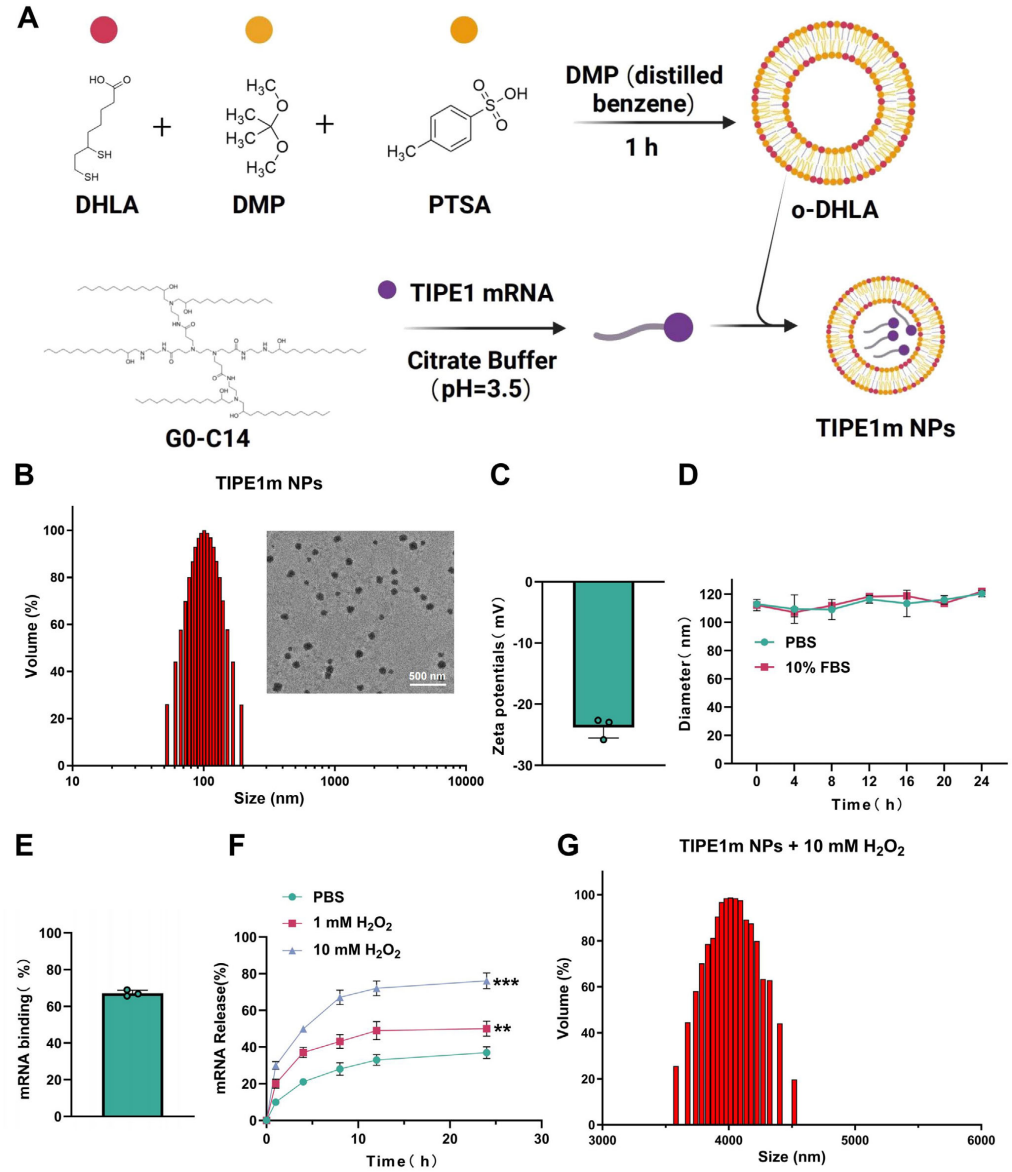
Construction and characterization of TIPE1m NPs. (A) Schematic illustration showing the design and mechanism of action of TIPE1m NPs; (B) DLS analysis of particle size and zeta potential of TIPE1m NPs, with inset showing TEM imaging of nanoparticle morphology (scale bar = 500 nm); (C) Zeta potential of TIPE1m NPs measured by DLS; (D) Stability of TIPE1m NPs after 48‐h incubation in PBS containing 10% FBS, assessed by DLS; (E) mRNA EE% of TIPE1m NPs; (F) mRNA release profile of TIPE1m NPs under different H_2_O_2_ concentrations (0, 1 mm, and 10 mm) at 0, 1, 4, 8, 12, and 24 h. Data are presented as mean ± SD, *n* = 3 independent samples per group/time point. Statistical analysis was performed using two‐way ANOVA followed by Šídák's multiple comparisons test. The treatment effect, time effect, and treatment × time interaction were all significant (treatment: *p* = 2.91 × 10^−^^2^ ^5^; time: *p* = 1.76 × 10^−^^3^ ^1^; interaction: *p* = 1.93 × 10^−^^1^ ^2^). Exact adjusted *p* values for pairwise comparisons at each time point are provided in the legend/Supporting Tables; (G) Changes in particle size of TIPE1m NPs before and after H_2_O_2_ treatment, measured by DLS. All data are presented as mean ± SD. Experiments were performed in triplicate. Statistical analysis was conducted using ANOVA followed by Tukey's post hoc test. **p < 0.01, ***p < 0.001, ****p < 0.0001.

Given the elevated intracellular ROS levels in TNBC cells, we developed ROS‐responsive TIPE1m NPs for targeted gene delivery. Because TIPE1 overexpression was associated with ubiquitination‐related RAB7A reduction, impaired autophagic flux, increased mitochondrial ROS accumulation, and enhanced PTX‐induced apoptosis, TIPE1m NPs were designed to release TIPE1 mRNA in high‐ROS environments and thereby amplify ROS‐mediated apoptotic signaling (Figure 6A).

We first synthesized a ROS‐degradable polymer (o‐DHLA) through a condensation reaction between DHLA and DMP. Using a self‐assembly strategy, we then complexed TIPE1 mRNA with the cationic lipid G0‐C14 to form a stable core encapsulated with o‐DHLA, followed by surface modification with DSPE‐PEG to improve colloidal stability and biocompatibility. DLS measurements showed that the TIPE1m NPs had a mean particle size of approximately 100 nm and a zeta potential of −23.80 ± 1.74 mV (Figure 6B,C), suitable for efficient cellular uptake and systemic stability. TEM revealed that the nanoparticles exhibited uniform spherical morphology with smooth surfaces and no obvious aggregation (Figure 6B). After incubation in PBS containing 10% FBS at 37°C for 48 h, particle size increased by less than 10%, indicating good physiological stability (Figure 6D). Analysis of mRNA loading revealed that the EE% reached 66.2% ± 2.5% (Figure 6E).

To assess the ROS‐responsive behavior of the nanoparticles, we incubated them in PBS (control), 1 mM H_2_O_2_, and 10 mM H_2_O_2_ solutions, followed by quantification of mRNA release. In the PBS control group, mRNA release remained minimal over 24 h, whereas a significant increase in release was observed under 10 mm H_2_O_2_ treatment (Figure 6F). Furthermore, DLS analysis showed a marked increase in particle size after H_2_O_2_ exposure (Figure 6G), further confirming their ROS‐triggered degradability.

Together, these results demonstrate the successful construction of stable ROS‐responsive TIPE1m NPs with efficient mRNA encapsulation and ROS‐triggered release properties.

### 3.8 TIPE1M NP Enhances PTX Sensitivity in Drug‐Resistant TNBC Cells

As TIPE1m NPs are a ROS‐triggered mRNA delivery system, their release and function depend on intracellular ROS levels. Under PTX‐induced oxidative conditions, TIPE1m NPs can more effectively release TIPE1 mRNA, thereby enhancing TNBC cell sensitivity to PTX. To verify this hypothesis, we treated MDA‐MB‐231R cells with PTX to elevate intracellular ROS and assessed TIPE1 mRNA release and expression. DCFH‐DA fluorescence probe analysis showed that PTX significantly increased ROS levels, whereas pretreatment with the ROS scavenger NAC effectively suppressed PTX‐induced ROS elevation (Figure S12A). Subsequently, we used confocal laser scanning microscopy (CLSM) to visualize the cellular uptake and release of Cy5‐TIPE1 mRNA NPs. In PTX‐treated MDA‐MB‐231R cells, Cy5‐TIPE1 mRNA signals (red) were observed to relocate from lysosomes (LysoTracker Green, green) to the cytoplasm. In contrast, NAC pretreatment led to retention of mRNA within lysosomes (Figure S12B), indicating that PTX‐induced ROS promotes nanoparticle degradation and triggers TIPE1 mRNA release.

**FIGURE 7:**
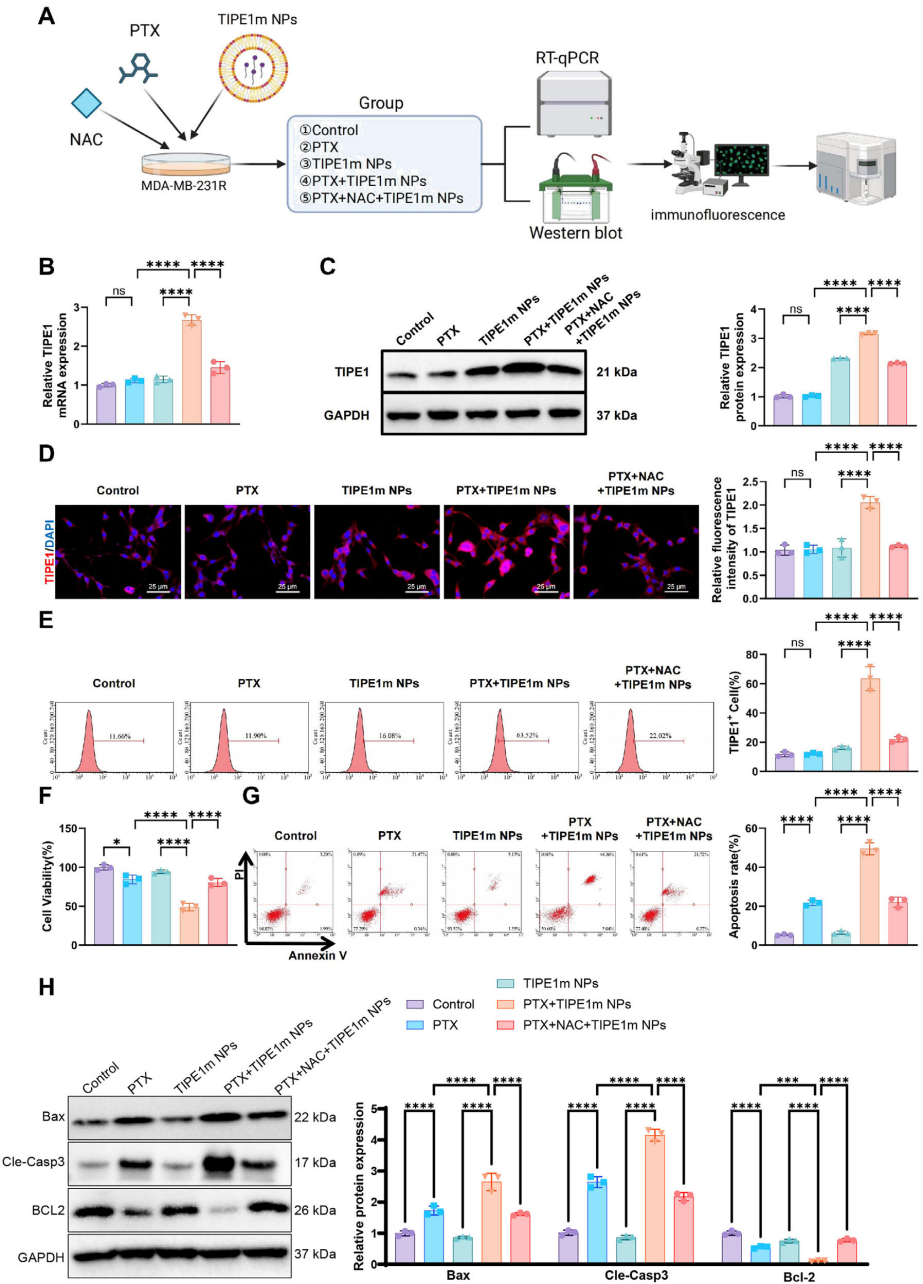
TIPE1m NPs enhances PTX‐induced TIPE1 expression and apoptotic effects. (A) Schematic workflow illustrating the combined treatment strategy using PTX, TIPE1m NPs, and NAC in MDA‐MB‐231R cells; (B,C) RT‐qPCR and WB analyses of TIPE1 mRNA and protein expression across groups; (D) Immunofluorescence staining to assess TIPE1 protein expression and intracellular localization (scale bar = 50 µm); (E) Flow cytometry analysis of TIPE1‐positive cell populations; (F) CCK‐8 assay to evaluate cell viability; (G) Annexin V/PI flow cytometry to determine apoptotic cell percentages; (H) WB analysis of apoptosis‐related proteins Bax, cleaved caspase‐3, and Bcl‐2. All data are presented as mean ± SD. Experiments were independently performed three times. Statistical comparisons were made using ANOVA followed by Tukey's post hoc test. *p < 0.05, **p < 0.01, ***p < 0.001, ****p < 0.0001.

To systematically evaluate the ROS‐responsive release of TIPE1m NPs, restoration of TIPE1 expression, and enhancement of PTX sensitivity in TNBC, we performed a series of experiments (Figure 7A). RT‐qPCR and WB analyses (Figure 7B,C) showed that TIPE1m NPs alone did not significantly increase TIPE1 expression, suggesting that the delivery system remains “off” under low ROS conditions. However, co‐treatment with PTX and TIPE1m NPs markedly upregulated both TIPE1 mRNA and protein levels, indicating that PTX‐induced ROS activated the mRNA release function of TIPE1m NPs. NAC pretreatment significantly inhibited this expression, confirming that TIPE1m NPs act as a ROS‐responsive delivery system. Immunofluorescence staining (Figure 7D) revealed the strongest TIPE1 signal in the PTX+TIPE1m NPs group, while the Control, PTX, and TIPE1m NPs groups showed weak expression; NAC pretreatment markedly suppressed TIPE1 fluorescence. Flow cytometry analysis also confirmed the highest proportion of TIPE1‐positive cells in the combination group (Figure 7E), further emphasizing that mRNA release and translation depend on ROS levels.

CCK‐8 assays showed that PTX significantly reduced cell viability compared with the control group, whereas TIPE1m NPs alone had no obvious effect (Figure 7F). Notably, combined PTX and TIPE1m NP treatment further decreased cell viability, indicating that TIPE1 restoration enhanced PTX‐induced cytotoxicity. This effect was partially reversed by N‐acetylcysteine (NAC) pretreatment. Annexin V‐based flow cytometric apoptosis analysis (Figure 7G) further confirmed that PTX induced apoptosis, and the combination with TIPE1m NPs significantly increased the apoptotic ratio. In the PTX + NAC + TIPE1m NPs group, apoptosis was reduced, suggesting that ROS contributes to the pro‐apoptotic effect mediated by TIPE1. WB analysis (Figure 7H) showed that, compared to the Control group, PTX treatment upregulated the expression of pro‐apoptotic proteins Bax and cleaved caspase‐3, and downregulated Bcl‐2, indicating its antitumor activity. Co‐treatment with TIPE1m NPs further amplified these protein expression changes, suggesting that TIPE1 enhances PTX efficacy by promoting mitochondrial pathway‐mediated apoptosis. NAC pretreatment suppressed these expression trends.

In conclusion, the TIPE1m NPs we developed efficiently released TIPE1 mRNA under PTX‐induced high ROS conditions, restored its expression, and further enhanced PTX‐induced apoptotic effects.

### 3.9 In Vivo Validation of the Therapeutic Efficacy of TIPE1M NPs Against PTX Resistance

**FIGURE 8:**
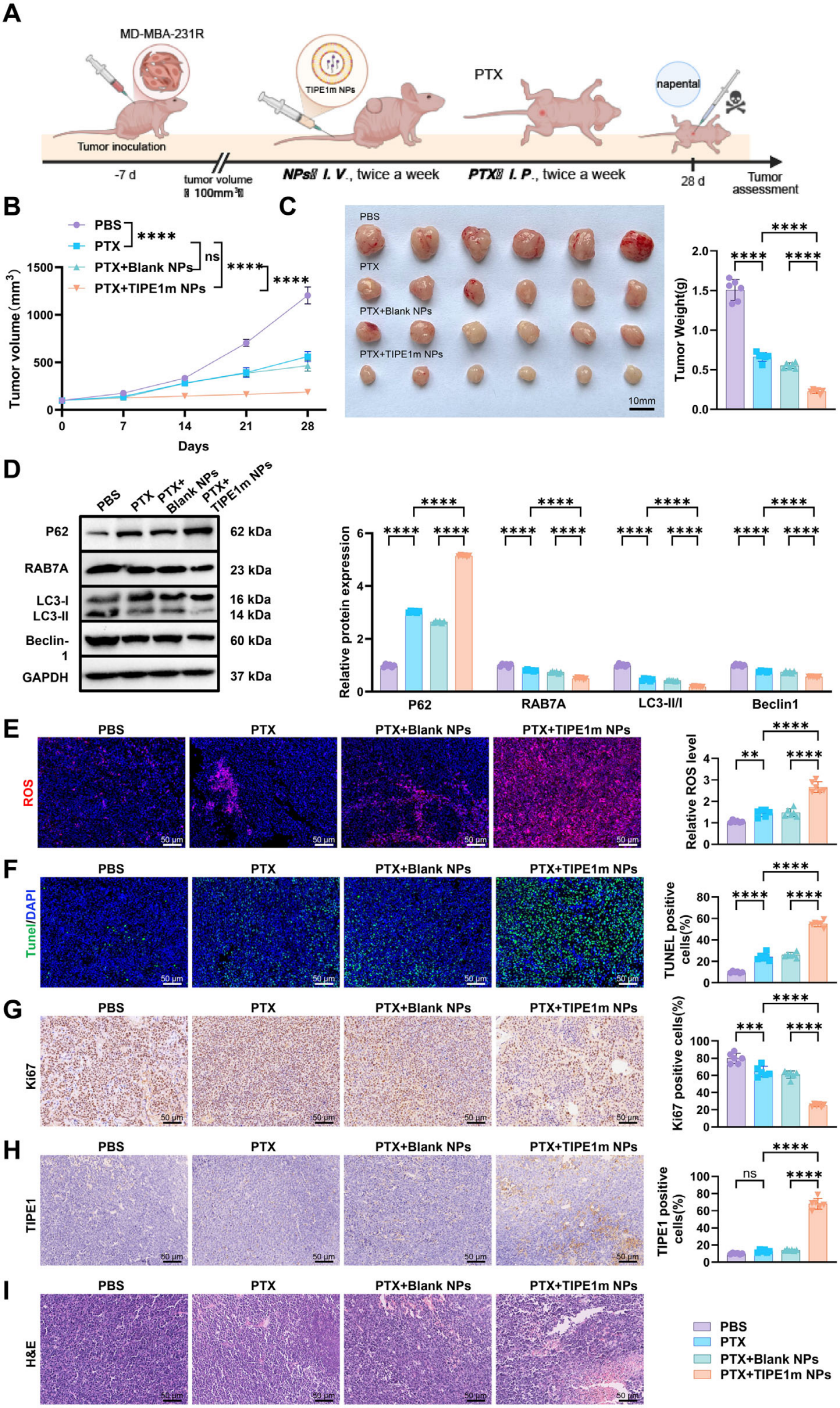
TIPE1m NPs combined with PTX markedly suppress MDA‐MB‐231R xenograft tumor growth and enhance apoptosis. (A) Schematic overview of the experimental design for establishing MDA‐MB‐231R subcutaneous tumor models and administering TIPE1m NPs and PTX; (B) Tumor volume monitoring to evaluate treatment efficacy across groups; (C) Representative tumor images and tumor weight measurements to assess growth inhibition; (D) WB analysis of RAB7A and autophagy‐related proteins LC3‐II/I, P62, and Beclin‐1 in tumor tissues; (E) DHE staining to detect ROS levels in tumor sections (scale bar = 50 µm); (F) TUNEL staining to assess apoptotic cells in tumor tissues (scale bar = 50 µm); (G) Ki67 IHC to evaluate tumor cell proliferation (scale bar = 50 µm); (H) TIPE1 IHC to detect TIPE1 expression in tumor tissues (scale bar = 50 µm); (I) H&E staining to observe histological architecture and cellular pathology in tumor sections (scale bar = 50 µm). All data are presented as mean ± SD, with *n* = 6 mice per group. Statistical analysis was performed using ANOVA followed by Tukey's post hoc test. *p < 0.05, **p < 0.01, ***p < 0.001, ****p < 0.0001, ns indicates no significant difference.

We established a subcutaneous xenograft model using PTX‐resistant MDA‐MB‐231R breast cancer cells and initiated treatment once tumor volumes reached approximately 100 mm^3^. Mice were randomly assigned to four groups: PBS, PTX, PTX + Blank NPs, and PTX + TIPE1m NPs. All groups received treatments twice per week as described in Methods, and animals were sacrificed on day 28 for histological and mechanistic analyses (Figure 8A). To evaluate the pharmacokinetics and intratumoral expression of TIPE1 delivered by TIPE1m NPs, we conducted plasma pharmacokinetic profiling and assessed TIPE1 expression in tumor tissues. Fluorescence tracking demonstrated that TIPE1m NPs significantly prolonged circulation time, with plasma fluorescence remaining detectable beyond 8 h (Figure S13A), supporting improved in vivo persistence after nanoparticle delivery. Furthermore, to assess the enhancement in stability and tumor‐targeting capacity conferred by the nanoparticle system, we intravenously injected nude mice bearing subcutaneous MDA‐MB‐231R tumors with either Cy5‐labeled free TIPE1 mRNA or Cy5‐TIPE1m NPs. Compared to the free mRNA group, Cy5 fluorescence accumulation was markedly increased in tumor tissues of the TIPE1m NPs group (Figure S13B), suggesting that the nanoparticle delivery platform effectively protects mRNA from enzymatic degradation in vivo and enhances tumor‐specific accumulation (Figure S13B).

Tumor volume was monitored during the 28‐day treatment period. Tumors in the PBS group grew rapidly, whereas PTX monotherapy only partially inhibited tumor progression. In contrast, tumor growth was markedly suppressed in the PTX + TIPE1m NPs group, with tumor volumes significantly lower than those in the other groups (Figure 8B). Tumor photographs and weight measurements further corroborated the volume data: tumors in the PTX + TIPE1m NPs group were the smallest and lightest, significantly outperforming both the PTX and PTX + Blank NPs groups, demonstrating that the nanoparticle platform substantially enhanced the antitumor efficacy of PTX (Figure 8C).

To explore the underlying mechanism, RAB7A and autophagy‐related proteins were examined in tumor tissues. WB showed that, compared with the PBS group, the PTX + TIPE1m NPs group had reduced RAB7A expression, LC3‐II/I ratio, and Beclin‐1 levels, together with marked p62 accumulation (Figure 8D). These results suggest that TIPE1m NPs inhibit autophagic flux in vivo, likely by promoting TIPE1‐mediated RAB7A degradation.

Next, we assessed ROS levels in tumor tissues to determine whether TIPE1m NPs influenced intracellular oxidative stress. DHE fluorescence staining revealed a markedly stronger ROS signal in the PTX + TIPE1m NPs group compared to the PTX and Blank NPs groups, indicating that TIPE1m NPs promoted mitochondrial ROS accumulation by blocking autophagy, thereby enhancing the cellular stress response (Figure 8E).

To evaluate whether TIPE1m NPs enhanced PTX‐induced apoptosis, TUNEL staining was performed on tumor tissues. The PTX + TIPE1m NPs group exhibited the highest proportion of TUNEL‐positive cells, significantly exceeding that of the PTX group. These findings suggest that the accumulation of ROS contributed to the potentiation of PTX‐induced apoptosis by TIPE1m NPs (Figure 8F).

IHC further supported these results. Ki‐67 staining revealed that the PTX + TIPE1m NPs group had the lowest proportion of Ki‐67‐positive cells, indicating a pronounced suppression of tumor cell proliferation by the combination treatment (Figure 8G). TIPE1 staining demonstrated significantly elevated TIPE1 expression in the PTX + TIPE1m NPs group, whereas other groups showed low or unchanged levels, suggesting that the nanoparticle system successfully released TIPE1m NPs in response to PTX‐induced ROS, thereby enhancing its intratumoral bioactivity (Figure 8H). H&E staining showed that tumors in the PBS group had densely packed cells with large, hyperchromatic nuclei. PTX treatment induced scattered necrosis, while the PTX + TIPE1m NPs group displayed extensive cellular degeneration, nuclear pyknosis, and architectural disruption, indicating substantial tissue damage caused by the combination therapy (Figure 8I).

Finally, long‐term biosafety was assessed in a 12‐week repeated‐dose observation study. No obvious body weight changes were observed among the treatment groups. Histological examination of the heart, liver, spleen, lung, and kidney revealed no apparent tissue injury or inflammation, and serum ALT, AST, BUN, and Cr levels showed no significant abnormalities (Figure S14A–C). These results indicate favorable preliminary in vivo safety of TIPE1m NPs under the tested conditions.

In summary, TIPE1m NPs effectively restored TIPE1 expression in vivo, promoted RAB7A degradation, blocked autophagic flux, induced ROS accumulation, and consequently suppressed the growth of drug‐resistant TNBC tumors while enhancing PTX‐induced apoptosis.

## 4 Discussion

PTX is commonly used for TNBC, but acquired resistance remains a major limitation [24]. PTX resistance involves multiple mechanisms, including drug efflux, microtubule‐associated alterations, non‐coding RNA regulation, epigenetic remodeling, autophagy, and the tumor microenvironment (TME) [25]. In this study, MDA‐MB‐231R and BT‐549R PTX‐resistant cell models were established by stepwise drug exposure, and TIPE1 was persistently downregulated in resistant cells. This is consistent with previous studies showing that TIPE1 participates in apoptosis, migration, and stress responses in several cancers [26, 27, 28]. Drug resistance induced by long‐term drug exposure is usually accompanied by multiple adaptive changes, among which ABC transporter‐mediated drug efflux is an important mechanism of taxane resistance. Increased ABCB1 expression and altered epigenetic status of its promoter have been reported to be associated with taxane resistance [29]. Therefore, TIPE1 downregulation is more likely to represent one relevant component of the PTX resistance network rather than a single determinant.

RAB7A is a key small GTPase involved in late‐stage autophagy, endolysosomal trafficking, and autophagosome–lysosome fusion [30]. In this study, TIPE1 overexpression was associated with reduced RAB7A protein levels, increased RAB7A ubiquitination, and altered autophagy‐related markers. CHX chase assays, MG132 treatment, and RAB7A‐K191R mutant experiments further supported ubiquitination‐related regulation of RAB7A stability. Public database and bioinformatic analyses also suggested that RAB7A contains multiple potential post‐translational modification sites and may be regulated by several E3 ubiquitin ligases or deubiquitinases. Given the reported involvement of RAB7A in tumor progression and drug response [31], our findings support a link between TIPE1 and RAB7A stability regulation. However, they do not determine whether TIPE1 directly mediates RAB7A ubiquitination through an enzymatic mechanism.

Autophagy‐related proteins have diverse roles in TNBC progression, stress adaptation, and treatment response [32]. By clearing damaged organelles and modulating oxidative stress, autophagy can promote tumor‐cell tolerance to chemotherapy. ROS homeostasis is also closely linked to cell death and therapeutic resistance in breast cancer, as redox alterations may affect apoptosis, autophagy, and other stress‐related fate programs [33]. In this study, TIPE1 knockdown increased autophagy‐related activity, reduced ROS levels, and attenuated PTX‐induced apoptosis, whereas TIPE1 overexpression reduced RAB7A levels, suppressed autophagy‐related processes, increased ROS accumulation, and enhanced apoptosis. Bafilomycin A1 weakened the increase in PTX IC_50_ caused by TIPE1 knockdown, supporting the involvement of autophagy in TIPE1‐mediated PTX response. Mito‐TEMPO further suggested that mitochondrial ROS contributes to TIPE1 overexpression‐induced ROS accumulation, although other ROS sources or redox regulatory mechanisms cannot be excluded.

For the delivery strategy, we developed ROS‐responsive TIPE1m NPs to restore TIPE1 expression in resistant TNBC cells. In vitro characterization demonstrated that TIPE1m NPs had nanoscale size, efficient mRNA encapsulation, and ROS‐triggered release properties. Cellular experiments further showed that TIPE1m NPs restored TIPE1 expression, reduced RAB7A levels, altered autophagy‐related processes, and enhanced PTX sensitivity. The ROS responsiveness of the o‐DHLA system may be attributed to DHLA/LA redox conversion; under oxidative conditions, DHLA forms disulfide structures, providing a chemical basis for ROS‐responsive release [34]. However, H_2_O_2_‐induced release experiments only simulate oxidative stress conditions and cannot fully represent the dynamic and heterogeneous ROS microenvironment within tumor tissues.

In vivo, combined treatment with TIPE1m NPs and PTX suppressed MDA‐MB‐231R xenograft growth, restored TIPE1 expression, reduced RAB7A levels, increased ROS signals, and enhanced apoptosis in tumor tissues. H&E staining of major organs, serum hepatic and renal function markers, and 12‐week repeated‐dose observation showed no obvious safety abnormalities, suggesting acceptable preliminary in vivo safety under the current conditions. Existing PTX resistance‐reversal strategies, including ABC transporter inhibition, autophagy modulation, and RNA‐based interventions, still face limitations in specificity, toxicity, or delivery efficiency [35, 36]. TIPE1m NPs may provide an alternative strategy by restoring a resistance‐associated molecule and modulating RAB7A‐related autophagy and ROS homeostasis. However, the current animal experiments used a cell line‐derived xenograft model, which cannot fully recapitulate TNBC heterogeneity, immune microenvironment, or clinical treatment context.

The upstream mechanism responsible for TIPE1 downregulation remains unclear. ZEB1 is involved in TNBC chemoresistance and epithelial‐state regulation and can regulate target gene transcription by binding E‐box sequences [37]. Given the TIPE1 downregulation observed in resistant cells, whether ZEB1 or other transcriptional or epigenetic regulators control TNFAIP8L1 expression warrants further investigation. However, promoter‐binding assays, chromatin accessibility profiling, transcription factor perturbation, and reporter assays were not performed in this study; therefore, this possibility remains to be explored.

The TNBC TME may also influence TIPE1‐associated resistance regulation. Immune cells, cytokines, and metabolic stress within the TNBC microenvironment are closely associated with chemoresistance and autophagy regulation [38]. In addition, TIPE1 has been reported to affect macrophage M2‐like activation through the PIP3/Akt/TGFβ axis [39]. However, the present study focused mainly on tumor‐cell‐intrinsic TIPE1–RAB7A‐related processes and did not systematically assess the effects of TIPE1m NPs on T cells, NK cells, macrophages, neutrophils, or cytokine profiles in the TME. PTX has been reported to affect tumor‐associated macrophage states and may enhance the efficacy of PD‐1/PD‐L1 blockade [40]. Therefore, the potential combination of TIPE1 restoration with immunotherapy or other agents requires further evaluation in immunocompetent models.

The molecular mechanism underlying TIPE1‐associated RAB7A ubiquitination remains incompletely defined. Ubiquitination regulates tumor biology by controlling protein stability, signal transduction, and cellular stress responses, and different ubiquitin chain types may lead to distinct protein fates [41]. In this study, PhosphoSitePlus analysis and RAB7A‐K191R mutant experiments suggested that K191 may contribute to ubiquitination‐related regulation of RAB7A. UbiBrowser and network analyses further indicated that SMURF1, SMURF2, NEDD4, HERC3, UBE3C, and STUB1 may be associated with RAB7A regulation. Recent studies also suggest that deubiquitination‐related networks can affect autophagy‐related cell death and therapeutic sensitivity in TNBC [42], while USP5‐mediated deubiquitination and substrate stabilization have been linked to TNBC progression [43]. These findings support the possibility that TIPE1 functions as a protein stability‐related regulatory factor. However, the E3 ligase directly responsible for RAB7A ubiquitination was not identified, and K48‐ and K63‐linked ubiquitin chains were not distinguished. Future studies involving candidate E3 screening, interaction validation, ubiquitin chain‐specific detection, and multi‐site mutagenesis are needed to clarify the molecular details of TIPE1–RAB7A ubiquitination regulation.

Several limitations should be acknowledged. First, the association between TNFAIP8L1 expression, breast cancer prognosis, and PTX response was mainly based on public datasets and was not validated in TNBC patient samples from this study. Future studies should integrate TNBC tissues, neoadjuvant chemotherapy response data, and immunohistochemistry/qPCR validation to clarify the clinical association between TIPE1 expression and PTX resistance. Second, the resistant models were generated mainly by long‐term PTX exposure, and resistance intensity or mechanisms may vary depending on the model construction strategy. Additional TNBC cell models, broader molecular subtypes, and patient‐derived models are needed for further validation. Third, the long‐term safety, immunogenicity, in vivo metabolism, recurrence‐suppressive effects, and efficacy of TIPE1m NPs under different dosing regimens require evaluation in more complex preclinical systems. Fourth, whether the TIPE1–RAB7A axis participates in tumor stemness, immune escape, or resistance to other chemotherapeutic agents remains to be determined.

Overall, our results suggest that TIPE1 downregulation is associated with increased RAB7A stability, autophagy‐related alterations, and ROS homeostasis remodeling in PTX‐resistant TNBC cells. Restoration of TIPE1 expression reduced RAB7A levels, altered autophagy‐related processes, increased ROS accumulation, and enhanced PTX‐induced apoptosis. ROS‐responsive TIPE1m NPs showed potential to improve PTX response both in vitro and in vivo, but further translation will require validation in patient‐derived and immune‐relevant models, together with long‐term safety and pharmacokinetic studies.

## 5 Conclusion

This study suggests that TIPE1 is associated with ubiquitination‐related regulation of RAB7A stability, which may contribute to autophagic flux inhibition, ROS accumulation, and enhanced PTX‐induced apoptosis in TNBC. These findings identify the TIPE1–RAB7A axis as an important regulator of PTX resistance. We further developed ROS‐responsive TIPE1m NPs to restore TIPE1 expression in resistant TNBC cells. TIPE1m NPs enhanced PTX sensitivity in vitro and suppressed resistant tumor growth in vivo, while showing favorable preliminary biosafety. Together, this study provides a potential therapeutic target and nanodelivery strategy for overcoming PTX resistance in TNBC. Nevertheless, further validation using patient‐derived models, immune‐relevant systems, long‐term toxicity assessment, and pharmacokinetic studies is needed before clinical translation. Future studies may also explore combination strategies with immunotherapy, PARP inhibitors, or other therapies for refractory TNBC.

## Author Contributions

Wei Hu, Qishuai Chen, Yan Ma, Yang Liu, Xianing Dong, Wenjuan Wei, Jianxin Du, Shusheng Qiu, Maojin Tian, and Peiqing Zhao contributed to the conception and design of the study, acquisition and analysis of data, interpretation of results, and manuscript revision. Wei Hu, Qishuai Chen, and Yan Ma performed the main experiments and data analysis. Yang Liu, Xianing Dong, and Wenjuan Wei contributed to cell, molecular, imaging, and animal experiments. Jianxin Du, Shusheng Qiu, Maojin Tian, and Peiqing Zhao supervised the study, provided resources, and revised the manuscript critically. All authors approved the final manuscript.

## Funding

This work was supported by the National Natural Science Foundation of China (Nos. 81972002, 12304241), Natural Science Foundation of Shandong Province (ZR2024MH175, ZR2023QC168, ZR2025MS1329), Taishan Young Scholar Foundation of Shandong Province (tsqnz20231257), Shandong Medical and Health Science and Technology Projects (202309030858), Beijing Vlove Charity Foundation‐Jingyi Research Fund Phase II (JVII2025‐0080226031), Shandong Provincial Medical Association (YXH2025YS043), and Zibo Medical and Health Science Research Projects (20231501093).

## Ethical Statement

All animal experiments were approved by the Animal Ethics Committee of Zibo Central Hospital Affiliated to Binzhou Medical University (No. 202212007).

## Conflicts of Interest

The authors declare no conflicts of interest.

## Supporting information




**Supporting File 1**: advs76975‐sup‐0001‐SuppMat.docx.

**Supporting File 2**: advs76975‐sup‐0002‐FigureS1‐S14.zip.

## Data Availability

All data generated or analyzed during this study are included in this article and/or its supplementary material files. Further enquiries can be directed to the corresponding author.
