# Supplementary material for: Nanoparticle‐Mediated TIPE1 mRNA Delivery Enhances Paclitaxel Sensitivity in Triple‐Negative Breast Cancer by Modulating RAB7A Ubiquitination‐Associated Stability and Autophagy
Source: Adv Sci (Weinh). 2026 Aug 6;13(60):e76975. doi: 10.1002/advs.76975 (PMC13445324; doi:10.1002/advs.76975)
Supplement: Supplementary file 1 [file ADVS-13-e76975-s002.docx]

**Supporting Information**

**Nanoparticle-mediated TIPE1 mRNA delivery enhances paclitaxel sensitivity in triple-negative breast cancer by modulating RAB7A ubiquitination-associated stability and autophagy**

**Supplementary Figure Legends**

**Figure S1. Analysis of drug sensitivity and apoptotic capacity in PTX-resistant TNBC cells.**

**Note:** (A) CCK-8 assays were performed to assess cell viability in MDA-MB-231 and MDA-MB-231R cells treated with different concentrations of PTX, and IC₅₀ values were calculated to evaluate drug sensitivity. (B) CCK-8 assays were performed to assess cell viability in BT-549 and BT-549R cells treated with different concentrations of PTX, and IC₅₀ values were calculated to evaluate drug sensitivity. (C) After 10 consecutive passages in drug-free medium, CCK-8 assays were performed to assess cell viability in MDA-MB-231, MDA-MB-231R, BT-549, and BT-549R cells under different PTX concentrations, and IC₅₀ values were calculated to evaluate the stability of the resistant phenotype. (D) Annexin V-FITC/PI flow cytometry was used to detect apoptosis in MDA-MB-231, MDA-MB-231R, BT-549, and BT-549R cells after treatment with 20 nM PTX. (E) Quantitative analysis of apoptosis rates in MDA-MB-231 and MDA-MB-231R cells. (F) Quantitative analysis of apoptosis rates in BT-549 and BT-549R cells. Data are presented as the mean ± SD from three independent experiments. Comparisons between two groups were performed using an unpaired two-tailed Student’s t-test; *****p* < 0.0001.

**Figure S2. Enrichment analysis of DEGs in PTX-resistant MDA-MB-231 cells.**

**Note:** (A) KEGG pathway enrichment analysis indicating DEG involvement in key signaling pathways; (B) Pathway network analysis mapping the interrelationships among enriched pathways and associated DEGs; (C) GO-BP analysis showing the enrichment of DEGs in biological regulatory processes; (D) GO-CC analysis demonstrating the subcellular localization patterns of DEGs; (E) GO-MF analysis revealing enrichment in regulatory and binding activities; (F) Integrated GO analysis summarizing DEG annotations across BP, CC, and MF categories with significance shown as -log_10_(*p*-value). All data are presented as mean ± SD. *p*-values were adjusted to control the FDR.

**Figure S3. GSEA reveals significantly enriched functional pathways in MDA-MB-231R cells.**

**Note:** GSEA was performed to evaluate the enrichment of DEGs between MDA-MB-231 and MDA-MB-231R cells in HALLMARK signaling pathways. The results showed significant enrichment of pathways associated with cell cycle and mitosis, including HALLMARK_MYC_TARGETS_V1, HALLMARK_G2M_CHECKPOINT, HALLMARK_E2F_TARGETS, and HALLMARK_MITOTIC_SPINDLE. In addition, enrichment of HALLMARK_CHOLESTEROL_HOMEOSTASIS suggested altered cholesterol regulation, while HALLMARK_UNFOLDED_PROTEIN_RESPONSE indicated an enhanced adaptive response to endoplasmic reticulum stress in drug-resistant cells. All analyses were conducted using the GSEA software, and pathway enrichment significance was evaluated based on the enrichment score and corresponding *p*-values.

**Figure S4. Identification of key genes associated with PTX resistance using LASSO and random forest algorithms.**

**Note:** (A-B) Random forest analysis ranked the importance of LASSO-selected feature genes based on MeanDecreaseAccuracy and MeanDecreaseGini, respectively. (C) Heatmap showing the normalized expression patterns of LASSO-selected genes in MDA-MB-231 and MDA-MB-231R cells. (D) ROC curve evaluating the classification performance of TNFAIP8L1 in distinguishing PTX-resistant states. All data are presented as mean ± SD. Feature selection and classification performance were assessed using cross-validation and appropriate statistical tests.

**Figure S5. Public database analysis of TNFAIP8L1 expression, prognosis, and PTX treatment response in breast cancer**
**Note:** (A) TNFAIP8L1 expression levels in normal breast tissues and breast cancer tissues were analyzed using the TCGA-BRCA dataset, with 292 normal samples and 1,099 tumor samples. (B) The association between TNFAIP8L1 expression and overall survival in patients with breast cancer was analyzed using the Kaplan–Meier Plotter database. (C) The association between TNFAIP8L1 expression and PTX treatment response in patients with breast cancer was analyzed using the ROC Plotter database. Comparisons between groups were analyzed using the default statistical methods of the corresponding databases or unpaired tests; *p < 0.05, ***p **< 0.001.**

**Figure S6. Validation of TIPE1 gene knockdown efficiency at mRNA and protein levels.**

**Note:** (A) RT-qPCR analysis of TIPE1 mRNA expression in MDA-MB-231 and BT-549 cells transduced with different shRNA constructs; (B) WB analysis of TIPE1 protein levels in MDA-MB-231 and BT-549 cells following knockdown by various shRNA sequences. All data are presented as mean ± SD. Experiments were repeated three times. Statistical analysis was performed using ANOVA followed by Tukey's post hoc test. ***p <* 0.01,****p <* 0.001, *****p <* 0.0001.

**Figure S7. oe-TIPE1 suppresses proliferation, migration, and invasion in PTX-resistant TNBC cells.**

**Note:** (A-B) CCK-8 assays assessing changes in PTX sensitivity after oe-TIPE1 in MDA-MB-231R and BT-549R cells; (C) Colony formation assay evaluating the impact of oe-TIPE1 on the proliferative capacity of resistant TNBC cells; (D) Wound healing assay measuring the effect of oe-TIPE1 on the migratory ability of resistant TNBC cells (bar = 100 μm); (E) Transwell assay analyzing the effect of oe-TIPE1 on the invasive capacity of resistant TNBC cells (bar = 50 μm). All data are presented as mean ± SD. Experiments were repeated three times. Statistical significance was determined using ANOVA followed by Tukey's post hoc test. **p <* 0.05, ***p <* 0.01, ****p <* 0.001, *****p <* 0.0001.

**Figure S8. PTX-resistant TNBC cells adapt to oxidative stress by enhancing autophagy and maintaining mitochondrial function.**

**Note:** (A) Schematic diagram outlining the integrated analysis strategy involving RT-qPCR, WB, autophagic flux assessment, and mitochondrial function evaluation; (B) RT-qPCR analysis of autophagy-related gene mRNA levels in MDA-MB-231, MDA-MB-231R, BT-549, and BT-549R cells; (C) WB analysis of autophagy-associated proteins LC3, Beclin-1, ATG5, and p62; (D) JC-1 staining for assessment of MMP (bar = 25 μm); (E) MitoTracker Red CMXRos staining to evaluate mitochondrial content (bar = 25 μm); (F) Quantification of MitoTracker fluorescence intensity; (G) DCFH-DA fluorescence probe detecting intracellular ROS levels in TNBC cells (bar = 25 μm); (H) Schematic model illustrating the adaptive mechanism by which PTX-resistant TNBC cells activate autophagic flux, enhance mitochondrial function, and scavenge ROS to cope with drug-induced oxidative stress. All data are presented as mean ± SD. Experiments were performed in triplicate. Statistical analysis was conducted using ANOVA followed by Tukey's post hoc test. ***p <* 0.01, *******p <* 0.0001.**

**Figure S9. Bafilomycin A1 intervention validates the mechanism by which TIPE1 regulates autophagy and mitochondrial function.**

**Note: (A) WB analysis of LC3, Beclin-1, ATG5, and p62 protein levels in MDA-MB-231 cells following Bafilomycin A1 treatment; (B) MDC staining to assess changes in autophagosome formation; (C) JC-1 staining to evaluate the effects of Bafilomycin A1 on MMP; (D) CCK-8 assay showing PTX IC₅₀ values in sh-NC and sh-TIPE1 MDA-MB-231 cells with or without Bafilomycin A1 treatment. All data are presented as mean ± SD. Experiments were repeated in triplicate. Statistical analysis was performed using ANOVA followed by Tukey's post hoc test. **p* < 0.05, ***p* < 0.01, ****p* < 0.001, *****p* < 0.0001.**

**Figure S10. Public database and bioinformatic analyses of the clinical relevance and ubiquitination-related regulatory network of RAB7A**
**Note:** (A) RAB7A expression levels in normal breast tissues and breast cancer tissues were analyzed using the TCGA-BRCA dataset, with 292 normal samples and 1,099 tumor samples. (B) The association between RAB7A expression and overall survival in patients with breast cancer was analyzed using the Kaplan–Meier Plotter database. (C) The association between RAB7A expression and PTX treatment response in patients with breast cancer was analyzed using the ROC Plotter database. (D) The distribution of post-translational modification sites in RAB7A was analyzed using the PhosphoSitePlus database. (E) Potential E3 ubiquitin ligases, DUBs, and related regulatory networks targeting RAB7A were predicted using the UbiBrowser database. (F) The functional association between TNFAIP8L1 and the RAB7A-related ubiquitination regulatory network was analyzed using NetworkAnalyst. Comparisons between groups were analyzed using the default statistical methods of the corresponding databases or unpaired tests; *p < 0.05, ***p < 0.001.

**Figure S11. Proteomic analysis of changes in the protein expression profile of resistant TNBC cells after TIPE1 overexpression**
**Note:** (A) TMT-based quantitative proteomic analysis was performed to assess the global protein expression profiles of MDA-MB-231R oe-NC and oe-TIPE1 cells, and principal component analysis (PCA) was used to evaluate group separation. (B) Volcano plot showing the number and distribution of differentially expressed proteins between MDA-MB-231R oe-NC and oe-TIPE1 cells. (C) Proteomic quantification of RAB7A protein expression in MDA-MB-231R oe-NC and oe-TIPE1 cells. (D) Proteomic quantification of the autophagy-related proteins ATG5 and BECN1 in MDA-MB-231R oe-NC and oe-TIPE1 cells. (E) KEGG pathway enrichment analysis showing enriched pathways among upregulated and downregulated differentially expressed proteins. (F) Proteomic quantification of Rab family GTPases in MDA-MB-231R oe-NC and oe-TIPE1 cells. Each group included three biological replicates. Comparisons between two groups were performed using an unpaired Student’s t-test; *p < 0.05, **p < 0.01, ***p < 0.001.

**Figure S12. PTX-induced ROS promotes mRNA release from TIPE1m NPs.**

**Note:** (A) DCFH-DA fluorescence staining to assess intracellular ROS levels in MDA-MB-231R cells following PTX and NAC treatment (scale bar = 25 μm); (B) CLSM to visualize intracellular distribution and lysosomal colocalization of Cy5-TIPE1 mRNA after treatment with TIPE1m NPs, using LysoTracker Green to label lysosomes (scale bar = 25 μm). All data are presented as mean ± SD. Experiments were independently repeated three times. Statistical analysis was performed using ANOVA followed by Tukey's post hoc test. *****p <* 0.0001.

**Figure S13. *In vivo* pharmacokinetics and tumor-targeting delivery efficiency of TIPE1m NPs.**

**Note: (A) Pharmacokinetic profile of TIPE1m NPs in mouse plasma determined by fluorescence tracing, with half-life calculation; (B) *In vivo* fluorescence imaging showing the biodistribution of Cy5-TIPE1m NPs versus free Cy5-TIPE1 mRNA in major organs and tumor tissues. All data are presented as mean ± SD, n = 6 mice per group. Statistical significance was determined by ANOVA followed by Tukey's post hoc test. **p* < 0.05, ***p* < 0.01, ****p* < 0.001, *****p* < 0.0001.**

**Figure S14. *In vivo* safety evaluation of TIPE1m NPs.**

**Note:** (A) H&E staining of major organs (heart, liver, spleen, lung, kidney) from mice in PBS, Blank NPs, and TIPE1m NPs groups to assess tissue histology (scale bar = 100 μm); (B) Body weight monitoring during treatment; (C) Serum biochemical assays evaluating liver (AST, ALT) and kidney (BUN, Cr) function to assess systemic toxicity of TIPE1m NPs. All data are presented as mean ± SD, n = 6 mice per group. Statistical analysis was performed using ANOVA followed by Tukey's post hoc test. ns indicates no statistically significant difference.

**Supplementary Tables**

**Table S1. RT-qPCR primer sequences.**

| **Gene** | **Forward Primer (5'-3')** | **Reverse Primer (5'-3')** |
| --- | --- | --- |
| Beclin-1 | GGGCTCCCGAGGGATGG | TTCCTCCTGGGTCTCTCCTG |
| ATG5 | GAGTAGTTGCCTGGAGGAGC | CATGTACCCCAAGAGGGACC |
| RAB7A | CAGACTGCTGCGTTCTGGTA | GCTTTGTGGCCACTTGTCTG |
| TIPE1 | CACCCAGACTGGCCCCTAAT | GGTGTCCATGGCCTTAGCAG |
| GAPDH | AATGGGCAGCCGTTAGGAAA | GCGCCCAATACGACCAAATC |

**Table S2. shRNA sequences.**

| **Name** | **Sequences (5'-3')** |
| --- | --- |
| sh-NC | TTCTCCGAACGTGTCACGT |
| sh-TIPE1-1 | CCAGAAGATGCTCAAGAACCT |
| sh-TIPE1-2 | GATGCTCAAGAACCTGGTCAA |

**Full Uncropped Gels and Blots**

Figure 1H-TIPE1

Figure 1H-GAPDH

Figure 3C-LC3-I LC3-II

Figure 3C-Beclin-1

Figure 3C-ATG5

Figure 3C-p62

Figure 3C-GAPDH

Figure 3C-pULK1

Figure 3C-ULK1

Figure 3E-LC3-I LC3-II

Figure 3E-Beclin-1

Figure 3E-ATG5

Figure 3E-p62

Figure 3E-GAPDH

Figure 4B-IP:HA-TIPE1

Figure 4B-IP:HA-Flag

Figure 4B-IP:IgG-TIPE1

Figure 4B-IP:IgG-Flag

Figure 4B-input-TIPE1

Figure 4B-input-RAB7A

Figure 4B-input-GAPDH

Figure 4C-IP-RAB7A

Figure 4C-IP-TIPE1

Figure 4C-Input-RAB7A

Figure 4C-Input-TIPE1

Figure 4C-Input-GAPDH

Figure 4D-RAB7A

Figure 4D-GAPDH

Figure 4D-TOMM20

Figure 4F-RAB7A

Figure 4F-GAPDH

Figure 4G-RAB7A

Figure 4G-GAPDH

Figure 4H-IP-His-Ub

Figure 4H-IP-Flag

Figure 4H-Input-RAB7A

Figure 4H-Input-TIPE1

Figure 4H-Input-GAPDH

Figure 4H-IP-His-Ub

Figure 4H-IP-Flag

Figure 4H-Input-RAB7A

Figure 4H-Input-TIPE1

Figure 4H-Input-GAPDH

Figure 5B-LC3-I-LC3-II

Figure 5B-p62

Figure 5B-GAPDH

Figure 5C-LC3-I-LC3-II

Figure 5C-p62

Figure 5C-GAPDH

Figure 5H-Bax

Figure 5H-Cle-Casp3

Figure 5H-BCL2

Figure 5H-GAPDH

Figure 5I-Bax

Figure 5I-Cle-Casp3

Figure 5I-BCL2

Figure 5I-GAPDH

Figure 7C-TIPE1

Figure 7C-GAPDH

Figure 7H-Bax

Figure 7H-Cle-Casp3

Figure 7H-BCL2

Figure 7H-GAPDH

Figure 8D-P62

Figure 8D-RAB7A

Figure 8D-LC3-I-LC3-II

Figure 8D-Beclin-1

Figure 8D-GAPDH

Figure S5B-MDA-MB-231(TIPE1)

Figure S5B-MDA-MB-231(GAPDH)

Figure S5B-BT-549(TIPE1)

Figure S5B-BT-549(GAPDH)

Figure S7C-LC3-I-LC3-II

Figure S7C-Beclin-1

Figure S7C-ATG5

Figure S7C-p62

Figure S7C-GAPDH

Figure S9A-LC3-I LC3-II

Figure S9A-Beclin-1

Figure S9A-ATG5

Figure S9A-p62

Figure S9A-GAPDH
