# Supplementary material for: Nanoparticle‐Mediated TIPE1 mRNA Delivery Enhances Paclitaxel Sensitivity in Triple‐Negative Breast Cancer by Modulating RAB7A Ubiquitination‐Associated Stability and Autophagy
Source: Adv Sci (Weinh). 2026 Aug 6;13(60):e76975. doi: 10.1002/advs.76975 (PMC13445324; doi:10.1002/advs.76975)
Supplement: Supplementary file 2 [file ADVS-13-e76975-s001.zip › Figure S1.pdf]

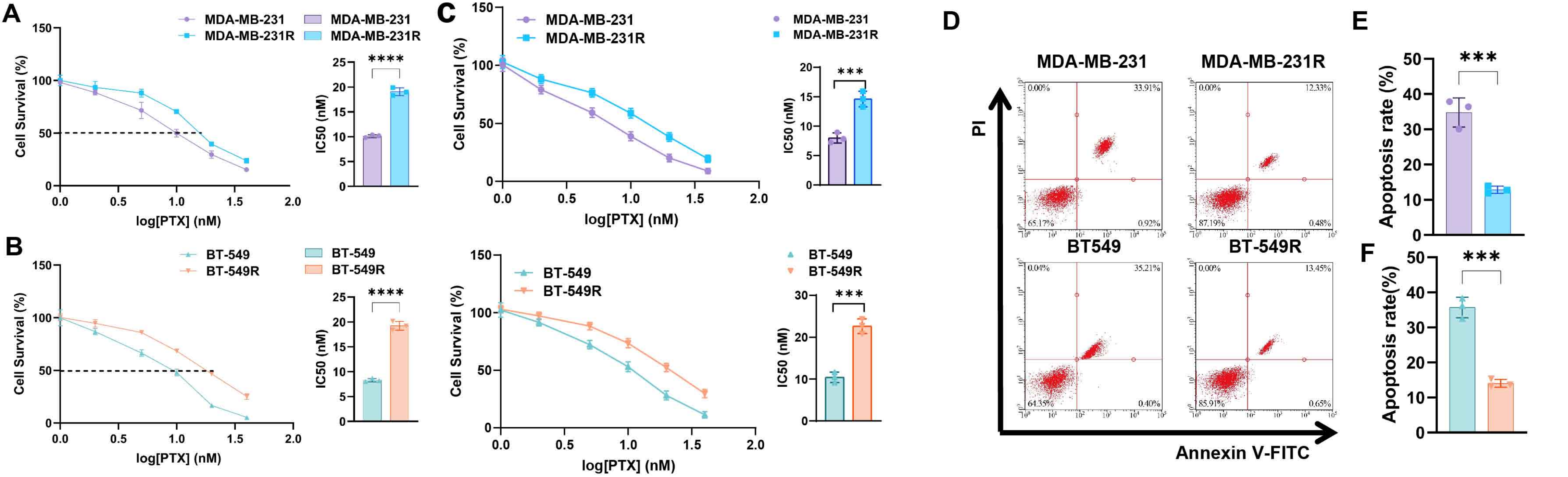
A
MDA-MB-231
MDA-MB-231R
Cell Survival (%)
log[PTX] (nM)
IC50 (nM)
****
B
BT-549
BT-549R
C
***
D
MDA-MB-231
MDA-MB-231R
BT549
BT-549R
PI
Annexin V-FITC
0.00%
33.91%
65.17%
0.92%
12.33%
87.19%
0.48%
0.04%
35.21%
64.35%
0.40%
13.45%
85.91%
0.65%
E
Apoptosis rate (%)
F
Apoptosis rate(%)
