# Supplementary material for: Nanoparticle‐Mediated TIPE1 mRNA Delivery Enhances Paclitaxel Sensitivity in Triple‐Negative Breast Cancer by Modulating RAB7A Ubiquitination‐Associated Stability and Autophagy
Source: Adv Sci (Weinh). 2026 Aug 6;13(60):e76975. doi: 10.1002/advs.76975 (PMC13445324; doi:10.1002/advs.76975)
Supplement: Supplementary file 2 [file ADVS-13-e76975-s001.zip › Figure S10.pdf]

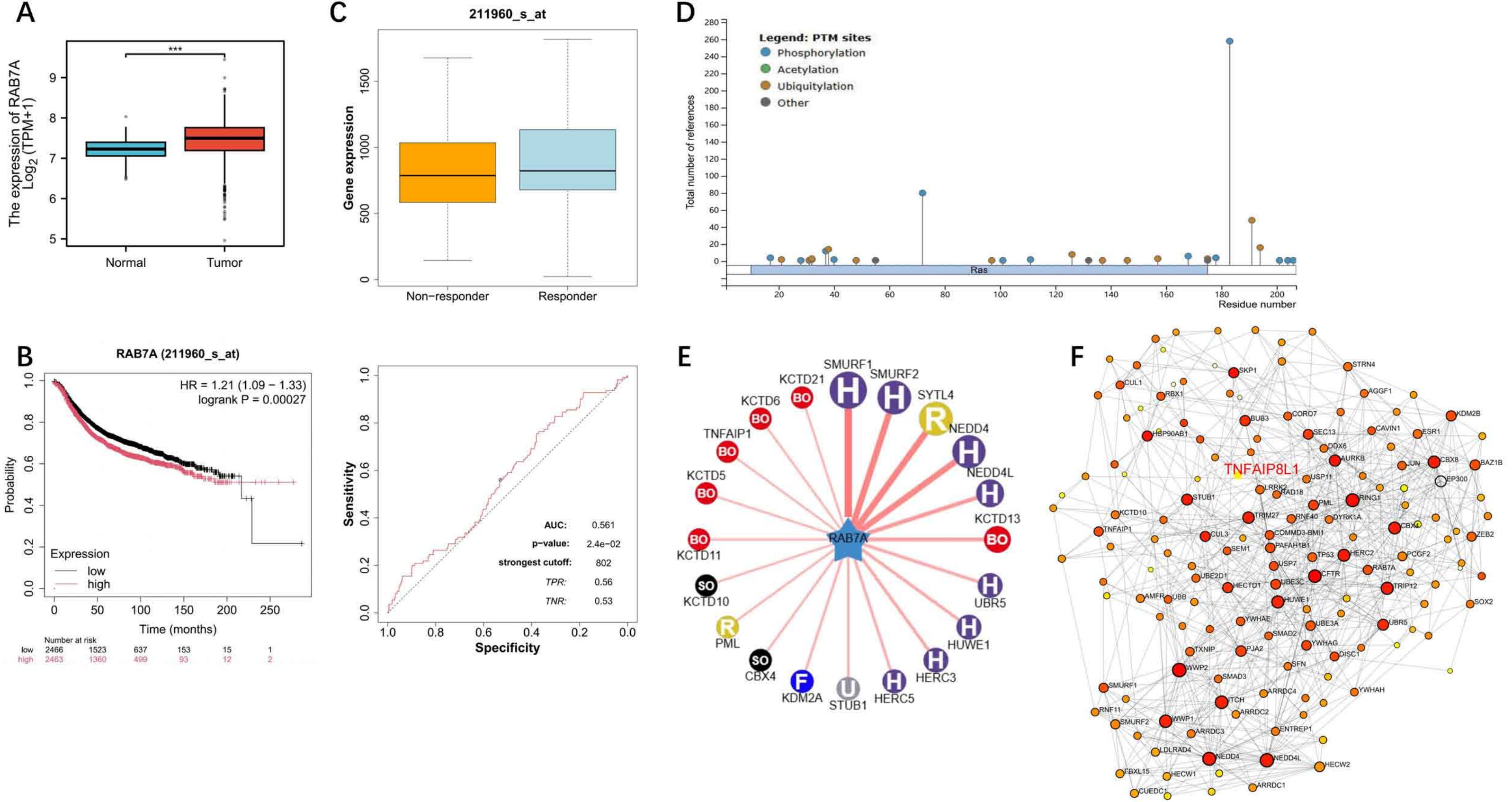
A
The expression of RAB7A Log2 (TPM+1)
***
Normal
Tumor
B
RAB7A (211960_s_at)
HR = 1.21 (1.09 − 1.33)
logrank P = 0.00027
Probability
Expression
low
high
Time (months)
Number at risk
low 2466 1523 637 153 15 1
high 2463 1360 499 93 12 2
C
211960_s_at
Gene expression
Non−responder
Responder
Sensitivity
Specificity
AUC: 0.561
p−value: 2.4e−02
strongest cutoff: 802
TPR: 0.56
TNR: 0.53
D
Legend: PTM sites
Phosphorylation
Acetylation
Ubiquitylation
Other
Total number of references
Ras
Residue number
E
SMURF1
SMURF2
SYTL4
NEDD4
NEDD4L
KCTD13
UBR5
HUWE1
HERC3
HERC5
STUB1
KDM2A
CBX4
PML
KCTD10
KCTD11
KCTD5
TNFAIP1
KCTD6
KCTD21
RAB7A
F
TNFAIP8L1
