# Supplementary material for: Nanoparticle‐Mediated TIPE1 mRNA Delivery Enhances Paclitaxel Sensitivity in Triple‐Negative Breast Cancer by Modulating RAB7A Ubiquitination‐Associated Stability and Autophagy
Source: Adv Sci (Weinh). 2026 Aug 6;13(60):e76975. doi: 10.1002/advs.76975 (PMC13445324; doi:10.1002/advs.76975)
Supplement: Supplementary file 2 [file ADVS-13-e76975-s001.zip › Figure S11.pdf]

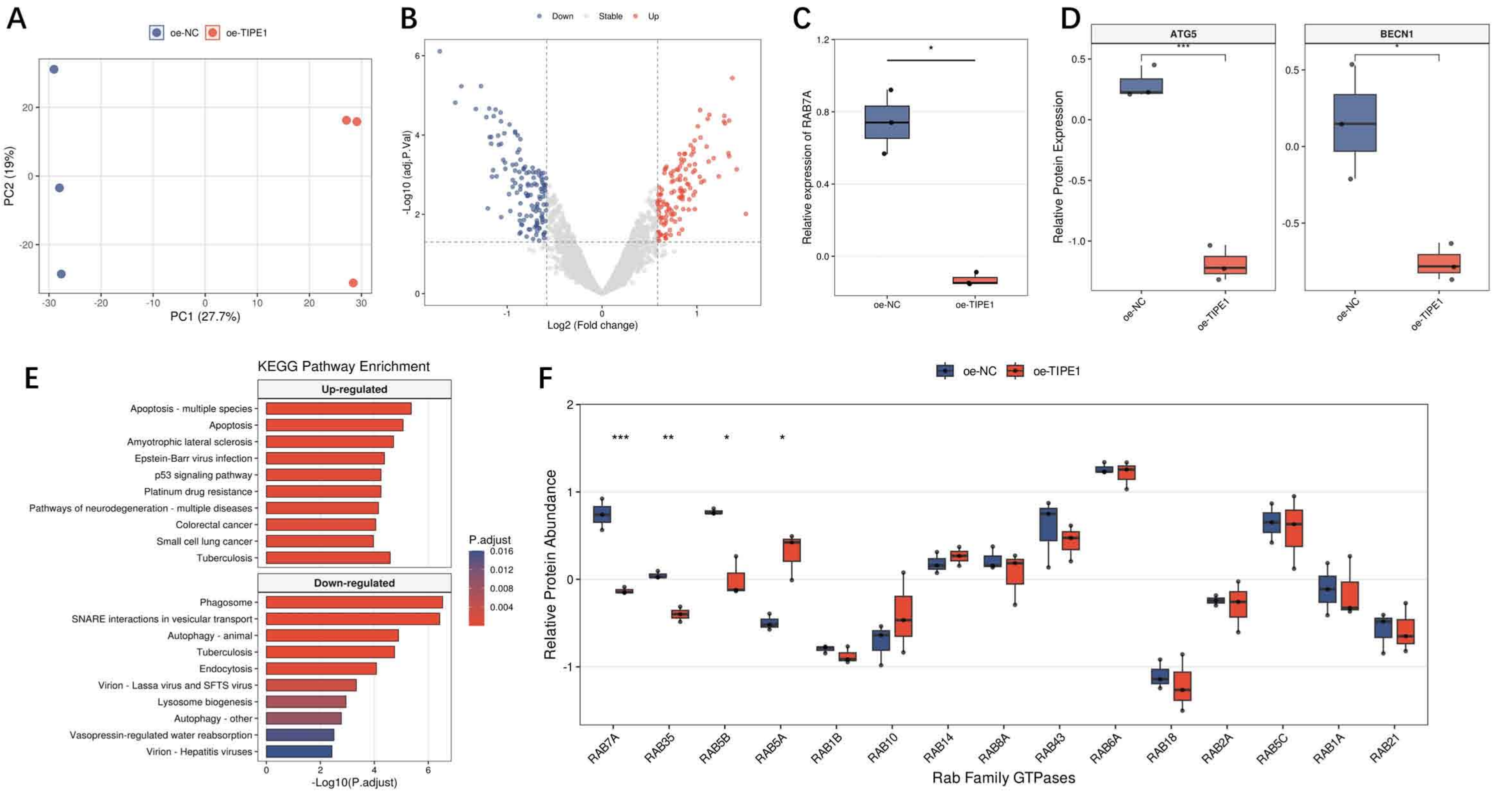

A
oe-NC
oe-TIPE1
PC1 (27.7%)
PC2 (19%)
B
Down
Stable
Up
Log2 (Fold change)
-Log10 (adj.P.Val)
C
Relative expression of RAB7A
oe-NC
oe-TIPE1
D
ATG5
BECN1
Relative Protein Expression
E
KEGG Pathway Enrichment
Up-regulated
Apoptosis - multiple species
Apoptosis
Amyotrophic lateral sclerosis
Epstein-Barr virus infection
p53 signaling pathway
Platinum drug resistance
Pathways of neurodegeneration - multiple diseases
Colorectal cancer
Small cell lung cancer
Tuberculosis
Down-regulated
Phagosome
SNARE interactions in vesicular transport
Autophagy - animal
Tuberculosis
Endocytosis
Virion - Lassa virus and SFTS virus
Lysosome biogenesis
Autophagy - other
Vasopressin-regulated water reabsorption
Virion - Hepatitis viruses
-Log10(P.adjust)
P.adjust
F
oe-NC
oe-TIPE1
Relative Protein Abundance
RAB7A
RAB35
RAB5B
RAB5A
RAB1B
RAB10
RAB14
RAB8A
RAB43
RAB6A
RAB18
RAB2A
RAB5C
RAB1A
RAB21
Rab Family GTPases
