# Supplementary material for: Nanoparticle‐Mediated TIPE1 mRNA Delivery Enhances Paclitaxel Sensitivity in Triple‐Negative Breast Cancer by Modulating RAB7A Ubiquitination‐Associated Stability and Autophagy
Source: Adv Sci (Weinh). 2026 Aug 6;13(60):e76975. doi: 10.1002/advs.76975 (PMC13445324; doi:10.1002/advs.76975)
Supplement: Supplementary file 2 [file ADVS-13-e76975-s001.zip › Figure S12.pdf]

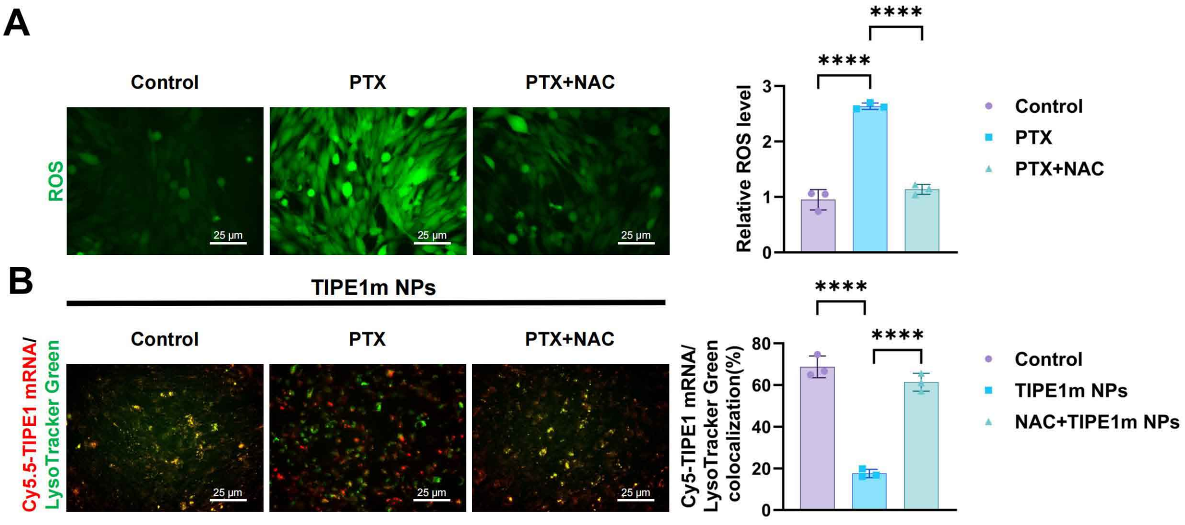
A
Control
PTX
PTX+NAC
ROS
25 μm
25 μm
25 μm
****
****
Relative ROS level
0
1
2
3
Control
PTX
PTX+NAC
B
TIPE1m NPs
Control
PTX
PTX+NAC
Cy5.5-TIPE1 mRNA/
LysoTracker Green
25 μm
25 μm
25 μm
****
****
Cy5-TIPE1 mRNA/
LysoTracker Green
colocalization(%)
0
20
40
60
80
Control
TIPE1m NPs
NAC+TIPE1m NPs
