# Supplementary material for: Nanoparticle‐Mediated TIPE1 mRNA Delivery Enhances Paclitaxel Sensitivity in Triple‐Negative Breast Cancer by Modulating RAB7A Ubiquitination‐Associated Stability and Autophagy
Source: Adv Sci (Weinh). 2026 Aug 6;13(60):e76975. doi: 10.1002/advs.76975 (PMC13445324; doi:10.1002/advs.76975)
Supplement: Supplementary file 2 [file ADVS-13-e76975-s001.zip › Figure S13.pdf]

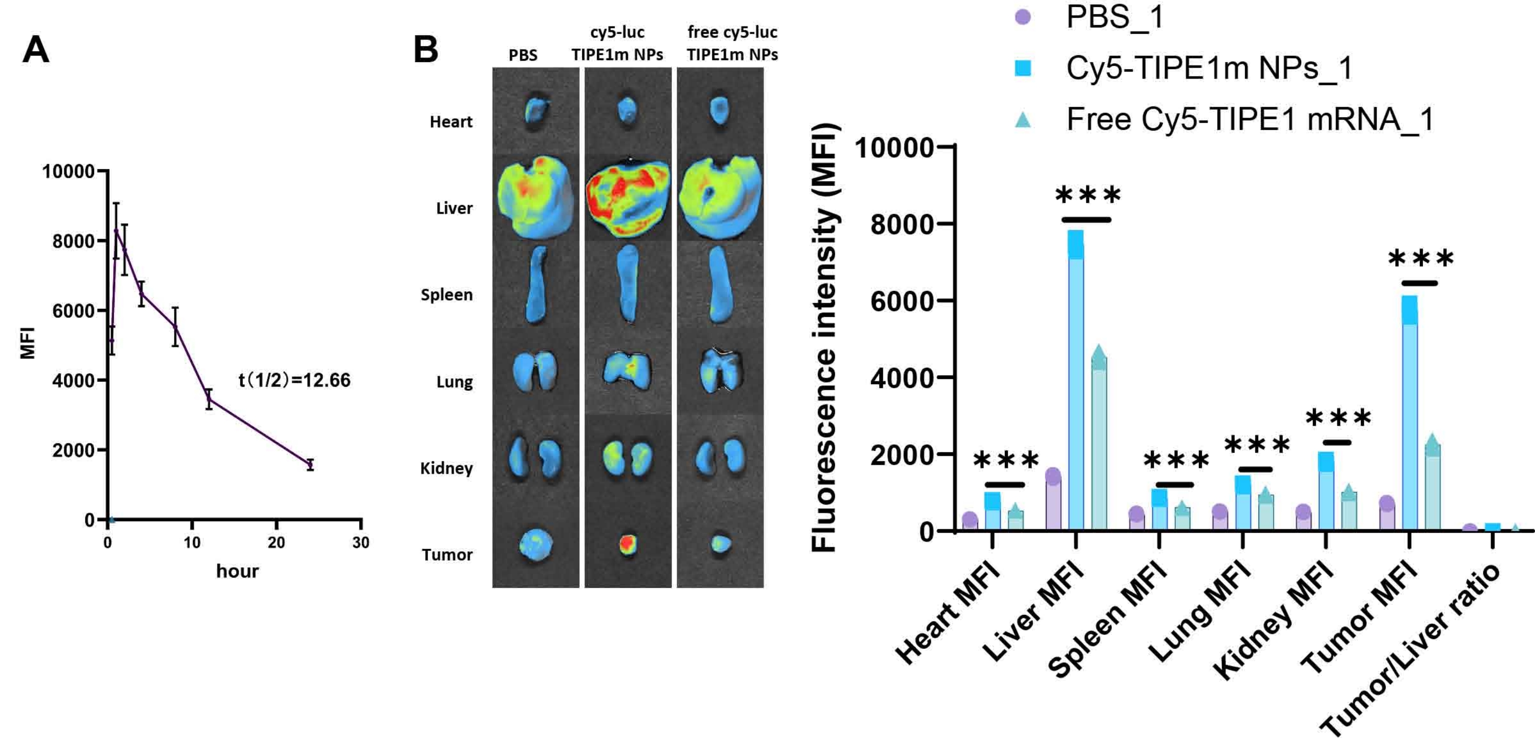
A
10000
8000
6000
4000
2000
0
MFI
t(1/2)=12.66
0
10
20
30
hour
B
PBS
cy5-luc TIPE1m NPs
free cy5-luc TIPE1m NPs
Heart
Liver
Spleen
Lung
Kidney
Tumor
PBS_1
Cy5-TIPE1m NPs_1
Free Cy5-TIPE1 mRNA_1
Fluorescence intensity (MFI)
10000
8000
6000
4000
2000
0
***
***
***
***
***
***
Heart MFI
Liver MFI
Spleen MFI
Lung MFI
Kidney MFI
Tumor MFI
Tumor/Liver ratio
