# Supplementary material for: Nanoparticle‐Mediated TIPE1 mRNA Delivery Enhances Paclitaxel Sensitivity in Triple‐Negative Breast Cancer by Modulating RAB7A Ubiquitination‐Associated Stability and Autophagy
Source: Adv Sci (Weinh). 2026 Aug 6;13(60):e76975. doi: 10.1002/advs.76975 (PMC13445324; doi:10.1002/advs.76975)
Supplement: Supplementary file 2 [file ADVS-13-e76975-s001.zip › Figure S14.pdf]

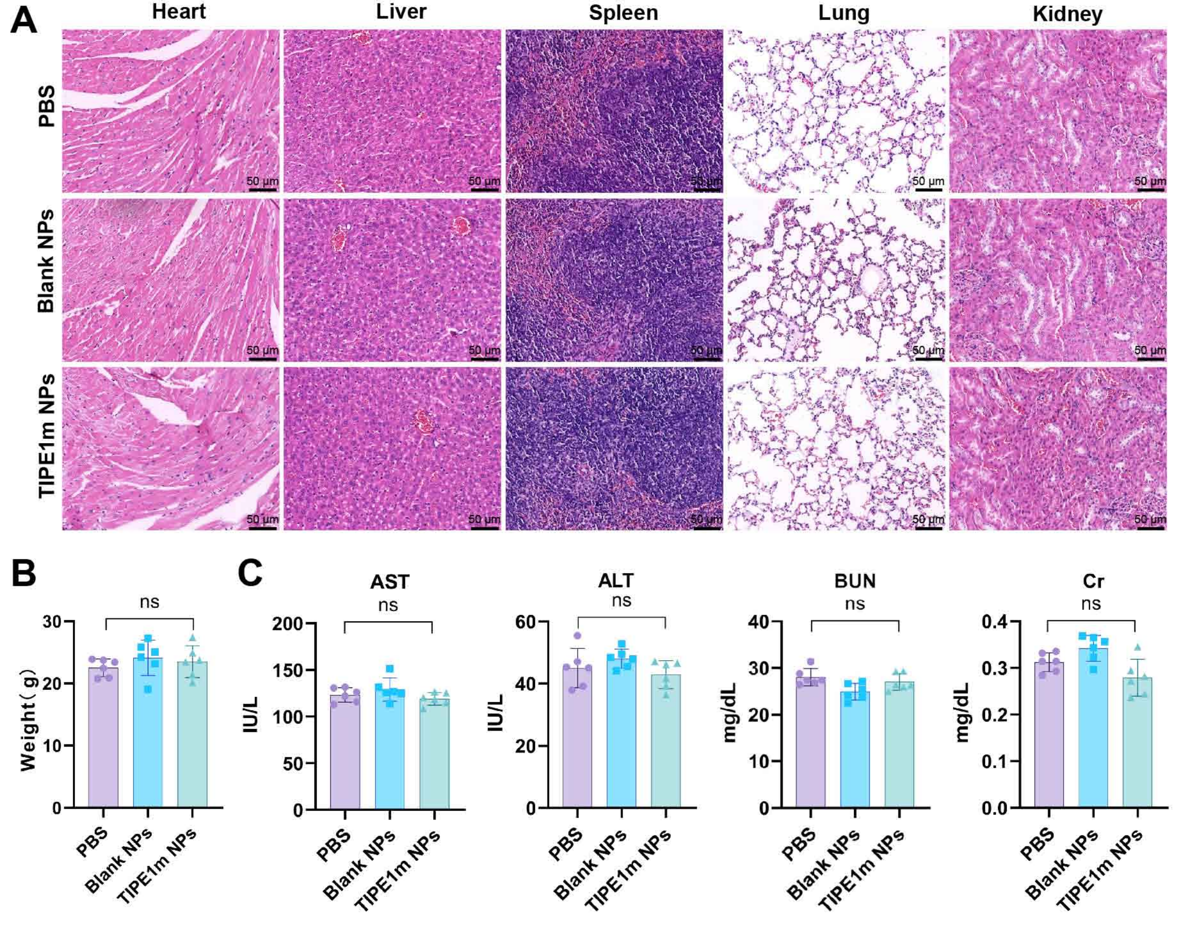
A
Heart
Liver
Spleen
Lung
Kidney
PBS
Blank NPs
TIPE1m NPs
50 μm
B
ns
Weight（g）
30
20
10
0
PBS
Blank NPs
TIPE1m NPs
C
AST
ns
IU/L
200
150
100
50
0
ALT
ns
IU/L
60
40
20
0
BUN
ns
mg/dL
40
30
20
10
0
Cr
ns
mg/dL
0.4
0.3
0.2
0.1
0.0
