# Supplementary material for: Nanoparticle‐Mediated TIPE1 mRNA Delivery Enhances Paclitaxel Sensitivity in Triple‐Negative Breast Cancer by Modulating RAB7A Ubiquitination‐Associated Stability and Autophagy
Source: Adv Sci (Weinh). 2026 Aug 6;13(60):e76975. doi: 10.1002/advs.76975 (PMC13445324; doi:10.1002/advs.76975)
Supplement: Supplementary file 2 [file ADVS-13-e76975-s001.zip › Figure S2.pdf]

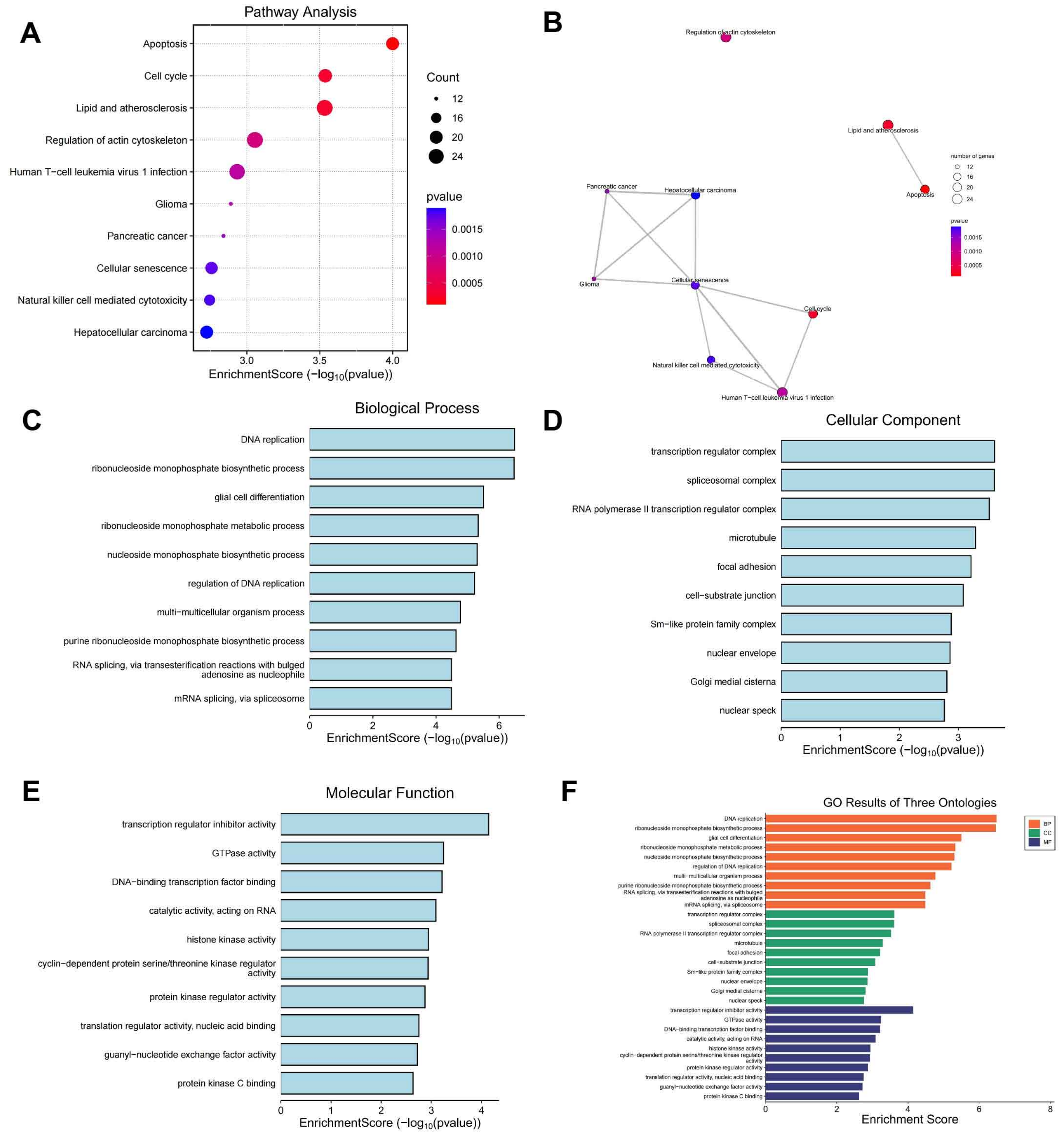
A
Pathway Analysis
Apoptosis
Cell cycle
Lipid and atherosclerosis
Regulation of actin cytoskeleton
Human T−cell leukemia virus 1 infection
Glioma
Pancreatic cancer
Cellular senescence
Natural killer cell mediated cytotoxicity
Hepatocellular carcinoma
3.0
3.5
4.0
EnrichmentScore (−log10(pvalue))
Count
12
16
20
24
pvalue
0.0015
0.0010
0.0005
B
Regulation of actin cytoskeleton
Lipid and atherosclerosis
Apoptosis
number of genes
12
16
20
24
pvalue
0.0015
0.0010
0.0005
Pancreatic cancer
Hepatocellular carcinoma
Glioma
Cellular senescence
Cell cycle
Natural killer cell mediated cytotoxicity
Human T−cell leukemia virus 1 infection
C
Biological Process
DNA replication
ribonucleoside monophosphate biosynthetic process
glial cell differentiation
ribonucleoside monophosphate metabolic process
nucleoside monophosphate biosynthetic process
regulation of DNA replication
multi−multicellular organism process
purine ribonucleoside monophosphate biosynthetic process
RNA splicing, via transesterification reactions with bulged adenosine as nucleophile
mRNA splicing, via spliceosome
0
2
4
6
EnrichmentScore (−log10(pvalue))
D
Cellular Component
transcription regulator complex
spliceosomal complex
RNA polymerase II transcription regulator complex
microtubule
focal adhesion
cell−substrate junction
Sm−like protein family complex
nuclear envelope
Golgi medial cisterna
nuclear speck
0
1
2
3
EnrichmentScore (−log10(pvalue))
E
Molecular Function
transcription regulator inhibitor activity
GTPase activity
DNA−binding transcription factor binding
catalytic activity, acting on RNA
histone kinase activity
cyclin−dependent protein serine/threonine kinase regulator activity
protein kinase regulator activity
translation regulator activity, nucleic acid binding
guanyl−nucleotide exchange factor activity
protein kinase C binding
0
1
2
3
4
EnrichmentScore (−log10(pvalue))
F
GO Results of Three Ontologies
BP
CC
MF
DNA replication
ribonucleoside monophosphate biosynthetic process
glial cell differentiation
ribonucleoside monophosphate metabolic process
nucleoside monophosphate biosynthetic process
regulation of DNA replication
multi−multicellular organism process
purine ribonucleoside monophosphate biosynthetic process
RNA splicing, via transesterification reactions with bulged adenosine as nucleophile
mRNA splicing, via spliceosome
transcription regulator complex
spliceosomal complex
RNA polymerase II transcription regulator complex
microtubule
focal adhesion
cell−substrate junction
Sm−like protein family complex
nuclear envelope
Golgi medial cisterna
nuclear speck
transcription regulator inhibitor activity
GTPase activity
DNA−binding transcription factor binding
catalytic activity, acting on RNA
histone kinase activity
cyclin−dependent protein serine/threonine kinase regulator activity
protein kinase regulator activity
translation regulator activity, nucleic acid binding
guanyl−nucleotide exchange factor activity
protein kinase C binding
0
2
4
6
8
Enrichment Score
