# Supplementary material for: Nanoparticle‐Mediated TIPE1 mRNA Delivery Enhances Paclitaxel Sensitivity in Triple‐Negative Breast Cancer by Modulating RAB7A Ubiquitination‐Associated Stability and Autophagy
Source: Adv Sci (Weinh). 2026 Aug 6;13(60):e76975. doi: 10.1002/advs.76975 (PMC13445324; doi:10.1002/advs.76975)
Supplement: Supplementary file 2 [file ADVS-13-e76975-s001.zip › Figure S3.pdf]

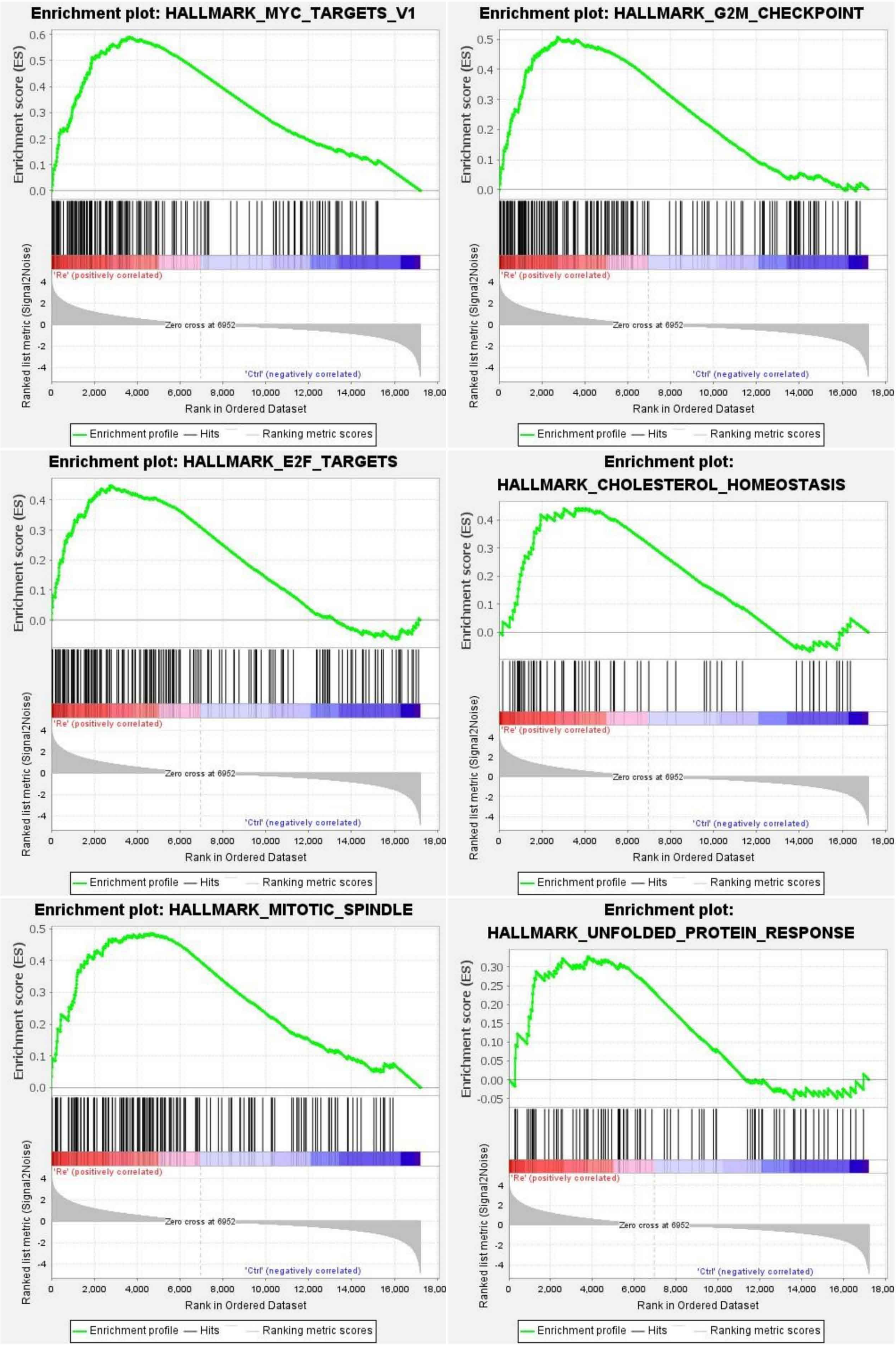

Enrichment plot: HALLMARK_MYC_TARGETS_V1
Enrichment plot: HALLMARK_G2M_CHECKPOINT
Enrichment plot: HALLMARK_E2F_TARGETS
Enrichment plot: HALLMARK_CHOLESTEROL_HOMEOSTASIS
Enrichment plot: HALLMARK_MITOTIC_SPINDLE
Enrichment plot: HALLMARK_UNFOLDED_PROTEIN_RESPONSE
Enrichment score (ES)
Ranked list metric (Signal2Noise)
'Re' (positively correlated)
Zero cross at 6952
'Ctrl' (negatively correlated)
Rank in Ordered Dataset
Enrichment profile
Hits
Ranking metric scores
