# Supplementary material for: Nanoparticle‐Mediated TIPE1 mRNA Delivery Enhances Paclitaxel Sensitivity in Triple‐Negative Breast Cancer by Modulating RAB7A Ubiquitination‐Associated Stability and Autophagy
Source: Adv Sci (Weinh). 2026 Aug 6;13(60):e76975. doi: 10.1002/advs.76975 (PMC13445324; doi:10.1002/advs.76975)
Supplement: Supplementary file 2 [file ADVS-13-e76975-s001.zip › Figure S4.pdf]

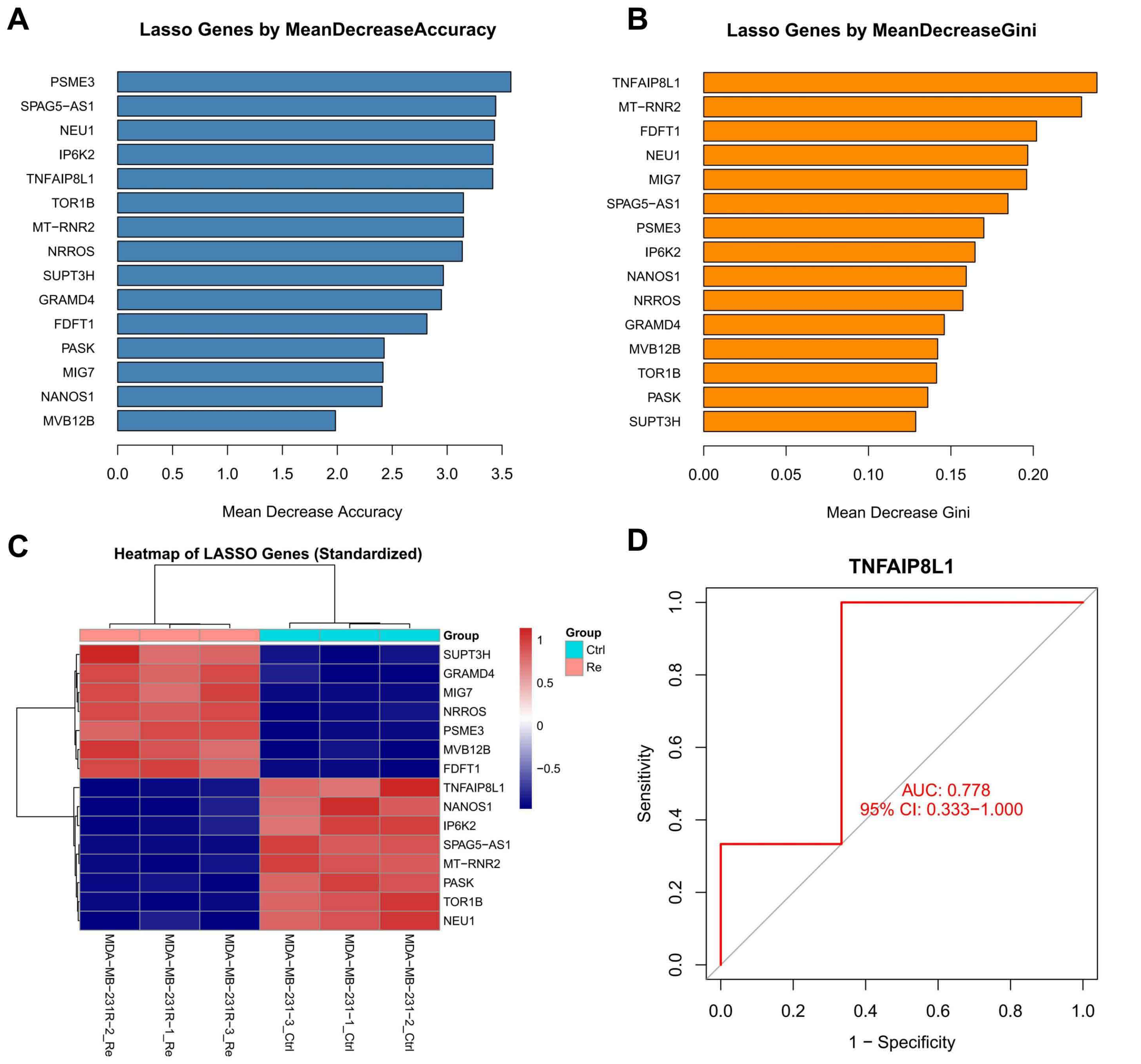
A
Lasso Genes by MeanDecreaseAccuracy
PSME3
SPAG5−AS1
NEU1
IP6K2
TNFAIP8L1
TOR1B
MT−RNR2
NRROS
SUPT3H
GRAMD4
FDFT1
PASK
MIG7
NANOS1
MVB12B
0.0 0.5 1.0 1.5 2.0 2.5 3.0 3.5
Mean Decrease Accuracy
B
Lasso Genes by MeanDecreaseGini
TNFAIP8L1
MT−RNR2
FDFT1
NEU1
MIG7
SPAG5−AS1
PSME3
IP6K2
NANOS1
NRROS
GRAMD4
MVB12B
TOR1B
PASK
SUPT3H
0.00 0.05 0.10 0.15 0.20
Mean Decrease Gini
C
Heatmap of LASSO Genes (Standardized)
Group
SUPT3H
GRAMD4
MIG7
NRROS
PSME3
MVB12B
FDFT1
TNFAIP8L1
NANOS1
IP6K2
SPAG5−AS1
MT−RNR2
PASK
TOR1B
NEU1
1
0.5
0
−0.5
Group
Ctrl
Re
MDA−MB−231R−2_Re
MDA−MB−231R−1_Re
MDA−MB−231R−3_Re
MDA−MB−231−3_Ctrl
MDA−MB−231−1_Ctrl
MDA−MB−231−2_Ctrl
D
TNFAIP8L1
Sensitivity
0.0 0.2 0.4 0.6 0.8 1.0
AUC: 0.778
95% CI: 0.333−1.000
1 − Specificity
0.0 0.2 0.4 0.6 0.8 1.0
