# Supplementary material for: Nanoparticle‐Mediated TIPE1 mRNA Delivery Enhances Paclitaxel Sensitivity in Triple‐Negative Breast Cancer by Modulating RAB7A Ubiquitination‐Associated Stability and Autophagy
Source: Adv Sci (Weinh). 2026 Aug 6;13(60):e76975. doi: 10.1002/advs.76975 (PMC13445324; doi:10.1002/advs.76975)
Supplement: Supplementary file 2 [file ADVS-13-e76975-s001.zip › Figure S5.pdf]

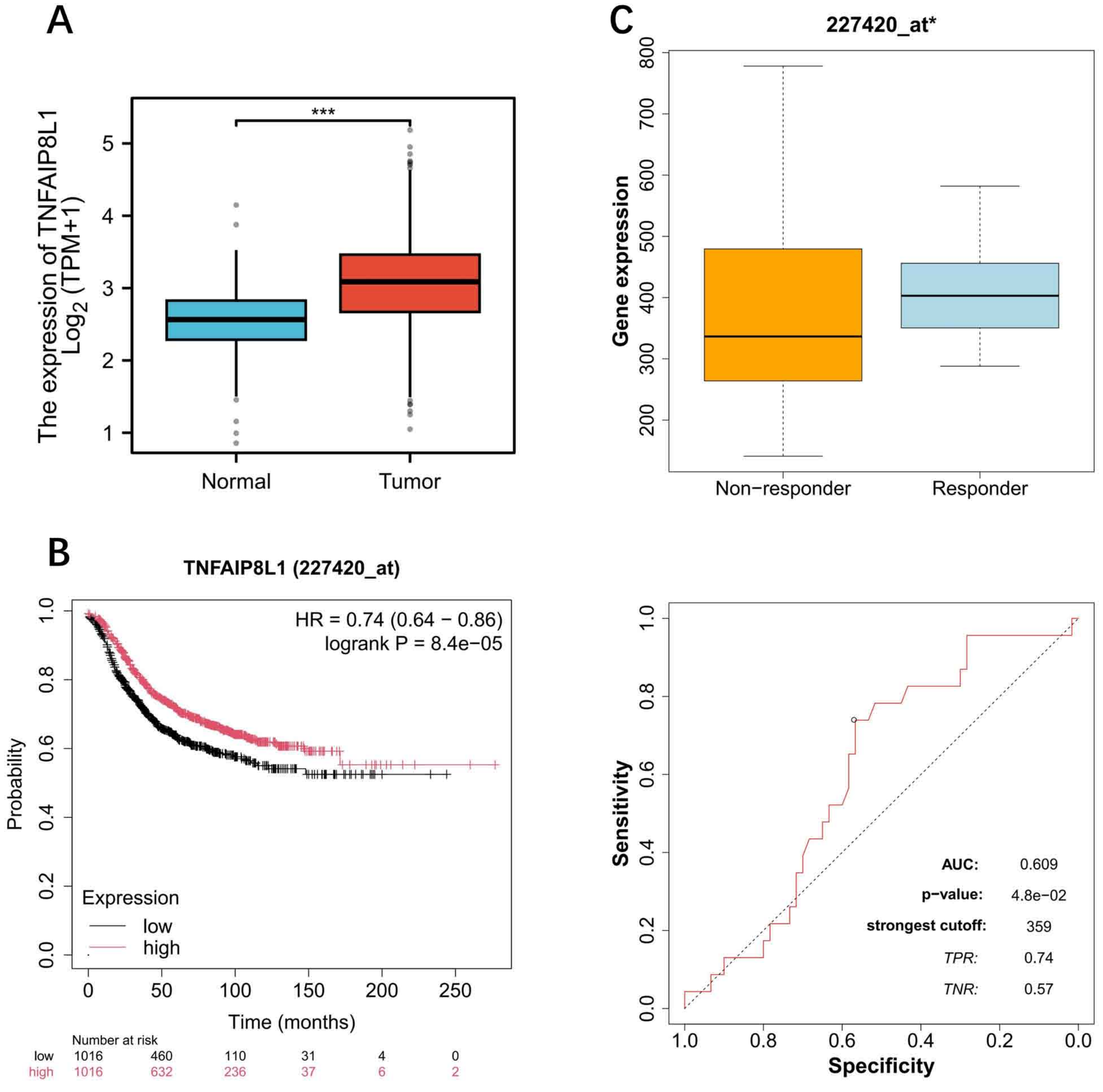
A
The expression of TNFAIP8L1
Log2 (TPM+1)
***
Normal
Tumor
B
TNFAIP8L1 (227420_at)
HR = 0.74 (0.64 − 0.86)
logrank P = 8.4e−05
Probability
Expression
low
high
Time (months)
Number at risk
low 1016 460 110 31 4 0
high 1016 632 236 37 6 2
C
227420_at*
Gene expression
Non−responder
Responder
Sensitivity
Specificity
AUC: 0.609
p−value: 4.8e−02
strongest cutoff: 359
TPR: 0.74
TNR: 0.57
