# Supplementary material for: Nanoparticle‐Mediated TIPE1 mRNA Delivery Enhances Paclitaxel Sensitivity in Triple‐Negative Breast Cancer by Modulating RAB7A Ubiquitination‐Associated Stability and Autophagy
Source: Adv Sci (Weinh). 2026 Aug 6;13(60):e76975. doi: 10.1002/advs.76975 (PMC13445324; doi:10.1002/advs.76975)
Supplement: Supplementary file 2 [file ADVS-13-e76975-s001.zip › Figure S6.pdf]

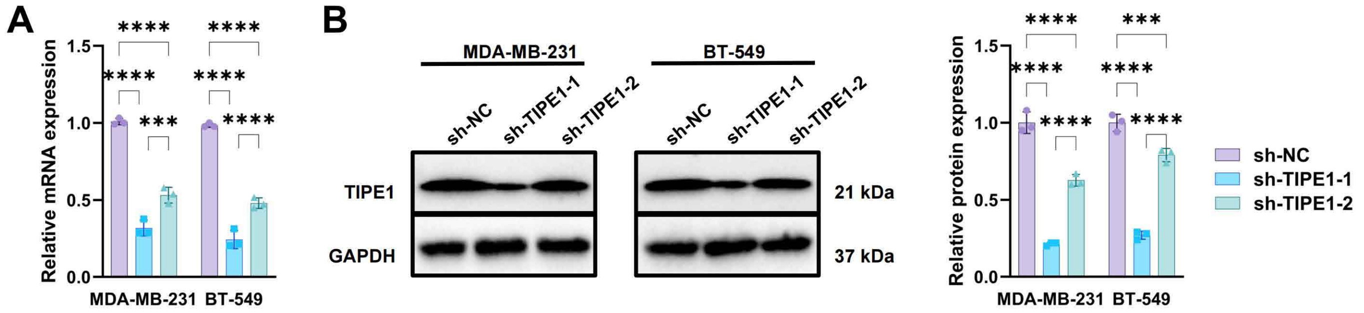

A
B
Relative mRNA expression
1.5
1.0
0.5
0.0
****
***
MDA-MB-231
BT-549
sh-NC
sh-TIPE1-1
sh-TIPE1-2
TIPE1
GAPDH
21 kDa
37 kDa
Relative protein expression
