# Supplementary material for: Nanoparticle‐Mediated TIPE1 mRNA Delivery Enhances Paclitaxel Sensitivity in Triple‐Negative Breast Cancer by Modulating RAB7A Ubiquitination‐Associated Stability and Autophagy
Source: Adv Sci (Weinh). 2026 Aug 6;13(60):e76975. doi: 10.1002/advs.76975 (PMC13445324; doi:10.1002/advs.76975)
Supplement: Supplementary file 2 [file ADVS-13-e76975-s001.zip › Figure S7.pdf]

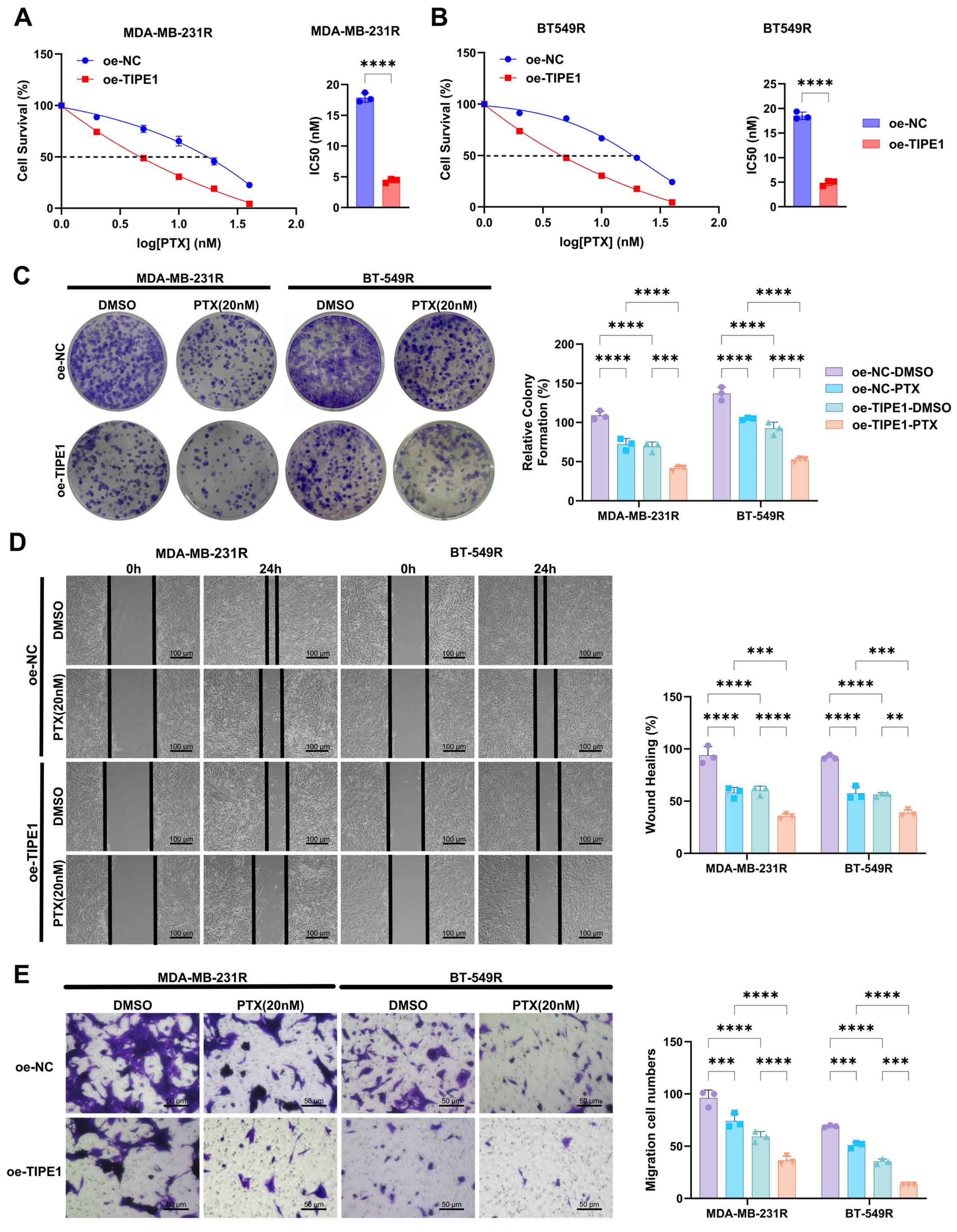
A
MDA-MB-231R
oe-NC
oe-TIPE1
Cell Survival (%)
log[PTX] (nM)
IC50 (nM)
****
B
BT549R
oe-NC
oe-TIPE1
Cell Survival (%)
log[PTX] (nM)
IC50 (nM)
****
C
MDA-MB-231R
BT-549R
DMSO
PTX(20nM)
oe-NC
oe-TIPE1
Relative Colony Formation (%)
oe-NC-DMSO
oe-NC-PTX
oe-TIPE1-DMSO
oe-TIPE1-PTX
D
MDA-MB-231R
BT-549R
0h
24h
DMSO
PTX(20nM)
oe-NC
oe-TIPE1
100 μm
Wound Healing (%)
E
MDA-MB-231R
BT-549R
DMSO
PTX(20nM)
oe-NC
oe-TIPE1
50 μm
Migration cell numbers
