# Supplementary material for: Nanoparticle‐Mediated TIPE1 mRNA Delivery Enhances Paclitaxel Sensitivity in Triple‐Negative Breast Cancer by Modulating RAB7A Ubiquitination‐Associated Stability and Autophagy
Source: Adv Sci (Weinh). 2026 Aug 6;13(60):e76975. doi: 10.1002/advs.76975 (PMC13445324; doi:10.1002/advs.76975)
Supplement: Supplementary file 2 [file ADVS-13-e76975-s001.zip › Figure S8.pdf]

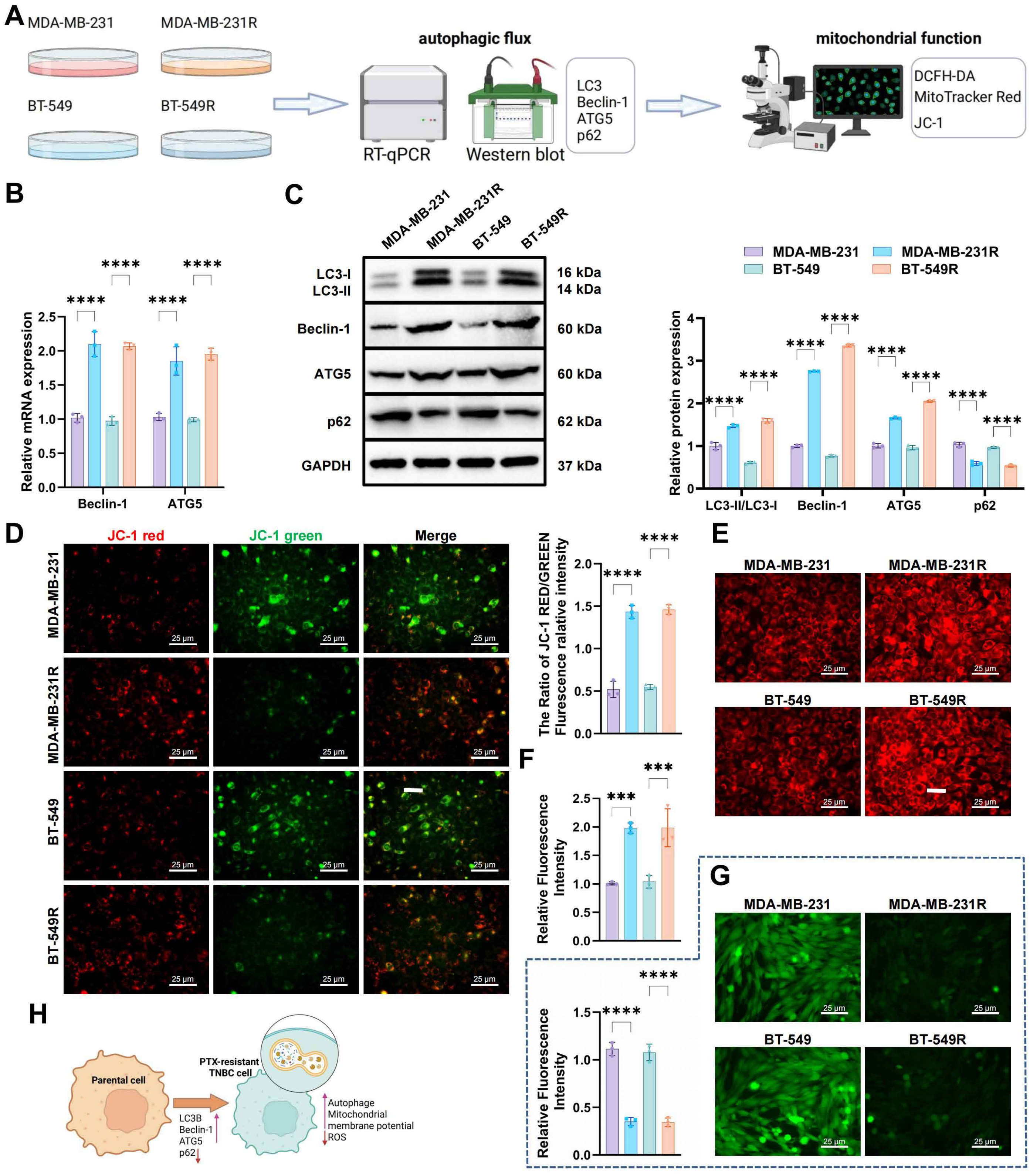
A
MDA-MB-231
MDA-MB-231R
BT-549
BT-549R
autophagic flux
RT-qPCR
Western blot
LC3
Beclin-1
ATG5
p62
mitochondrial function
DCFH-DA
MitoTracker Red
JC-1
B
Relative mRNA expression
Beclin-1
ATG5
C
LC3-I
LC3-II
Beclin-1
ATG5
p62
GAPDH
16 kDa
14 kDa
60 kDa
60 kDa
62 kDa
37 kDa
Relative protein expression
LC3-II/LC3-I
D
JC-1 red
JC-1 green
Merge
25 μm
The Ratio of JC-1 RED/GREEN Flurescence ralative intensity
E
F
Relative Fluorescence Intensity
G
H
Parental cell
PTX-resistant TNBC cell
LC3B
Beclin-1
ATG5
p62
Autophage
Mitochondrial membrane potential
ROS
